# The human long non-coding RNA gene *RMRP* has pleiotropic effects and regulates cell-cycle progression at G2

**DOI:** 10.1038/s41598-019-50334-6

**Published:** 2019-09-24

**Authors:** Svetlana Vakkilainen, Tiina Skoog, Elisabet Einarsdottir, Anna Middleton, Minna Pekkinen, Tiina Öhman, Shintaro Katayama, Kaarel Krjutškov, Panu E. Kovanen, Markku Varjosalo, Arne Lindqvist, Juha Kere, Outi Mäkitie

**Affiliations:** 1 Children’s Hospital, University of Helsinki and Helsinki University Hospital, Helsinki, Finland; 2 Folkhälsan Research Center, Institute of Genetics, Helsinki, Finland; 3 Department of Biosciences and Nutrition, Karolinska Institutet, Huddinge, Sweden; 4 Molecular Neurology Research Program, University of Helsinki, Helsinki, Finland; 5 Department of Cell and Molecular Biology, Karolinska Institutet, Stockholm, Sweden; 6 Institute of Biotechnology, and Helsinki Institute of Life Science, University of Helsinki, Helsinki, Finland; 7 Competence Centre on Health Technologies, Tartu, Estonia; 8 Department of Pathology, University of Helsinki, and HUSLAB, Helsinki University Hospital, Helsinki, Finland; 9 Department of Medical and Molecular Genetics, King’s College, London, UK; 10 Department of Molecular Medicine and Surgery, Karolinska Institutet and Clinical Genetics, Karolinska University Hospital, Stockholm, Sweden

**Keywords:** Immunological deficiency syndromes, Disease genetics

## Abstract

*RMRP* was the first non-coding nuclear RNA gene implicated in a disease. Its mutations cause cartilage-hair hypoplasia (CHH), an autosomal recessive skeletal dysplasia with growth failure, immunodeficiency, and a high risk for malignancies. This study aimed to gain further insight into the role of RNA Component of Mitochondrial RNA Processing Endoribonuclease (*RMRP*) in cellular physiology and disease pathogenesis. We combined transcriptome analysis with single-cell analysis using fibroblasts from CHH patients and healthy controls. To directly assess cell cycle progression, we followed CHH fibroblasts by pulse-labeling and time-lapse microscopy. Transcriptome analysis identified 35 significantly upregulated and 130 downregulated genes in CHH fibroblasts. The downregulated genes were significantly connected to the cell cycle. Multiple other pathways, involving regulation of apoptosis, bone and cartilage formation, and lymphocyte function, were also affected, as well as PI3K-Akt signaling. Cell-cycle studies indicated that the CHH cells were delayed specifically in the passage from G2 phase to mitosis. Our findings expand the mechanistic understanding of CHH, indicate possible pathways for therapeutic intervention and add to the limited understanding of the functions of *RMRP*.

## Introduction

Cartilage hair-hypoplasia (CHH, MIM #250250) is a rare autosomal recessive disorder characterized by metaphyseal chondrodysplasia leading to short stature (adult height on average 110–135 cm), hair hypoplasia, variable immunodeficiency, anemia, increased risk of malignancies, and Hirschsprung disease^1^. CHH is caused by mutations in *RMRP*, a gene that encodes a non-coding RNA, the RNA Component of Mitochondrial RNA Processing Endoribonuclease (*RMRP*)^2^. The founder mutation n.71 A > G (NCBI reference sequence: NR_003051.3) has been detected in almost all previously reported Finnish patients with CHH either in homozygous or heterozygous state^3^. This substitution affects evolutionary conserved nucleotides^4^, but the direct effect of this variant on the function of *RMRP* remains undescribed, and the variability of phenotype in patients with identical mutations is unexplained^1^.

Studies in yeast have shown that *RMRP* participates in mitochondrial DNA replication, rRNA processing and cell cycle progression at the end of mitosis^5^. Functional studies in humans have highlighted the role of *RMRP* in ribosomal assembly and cyclin A2 and B2-dependent cell cycle regulation^6^. Knockdown of *RMRP* in a normal human gastric mucosa epithelial cell line and gastric cancer cell lines resulted in cyclin D2 downregulation^7^. CRISPR disruption of *RMRP* in HeLa cells led to severe defects in cell proliferation and rRNA processing^8^.

Despite this work the specific pathogenesis of CHH remains incompletely understood. A generalized defect in cell proliferation has been speculated to explain several of the clinical manifestations, including disorganized growth-plate chondrocyte maturation, hair hypoplasia, immunodeficiency and impaired spermatogenesis^1,9,10^. Defective cell proliferation has been confirmed in T and B lymphocytes^9,11^, erythrocyte progenitors^12^ and fibroblasts^9^. Other studies have demonstrated increased apoptosis^13^ in CHH T lymphocytes, as well as a prolonged cell cycle with fewer cells entering S phase and a higher proportion of cells remaining in the G2/M phase^14^.

Most data on the function of *RMRP* and pathogenesis of CHH are inferred from experimental systems involving cancer cells and nonhuman cells. As many of the previous observations point toward a direct role of *RMRP* in ribosomal RNA processing and thus potentially transcriptome balance^5,6,8^, we assessed fibroblast transcriptomes in CHH patient cells and healthy control cells and followed up on suggested pathway effects by relevant functional assays. Our transcriptome data support a central role for *RMRP* in cell cycle regulation, mutations leading to slowing down of the process in CHH patients, supported by data from cell-cycle analysis in CHH fibroblasts. Other cellular systems relevant for the CHH phenotype were also affected.

## Results

**Figure 1:**
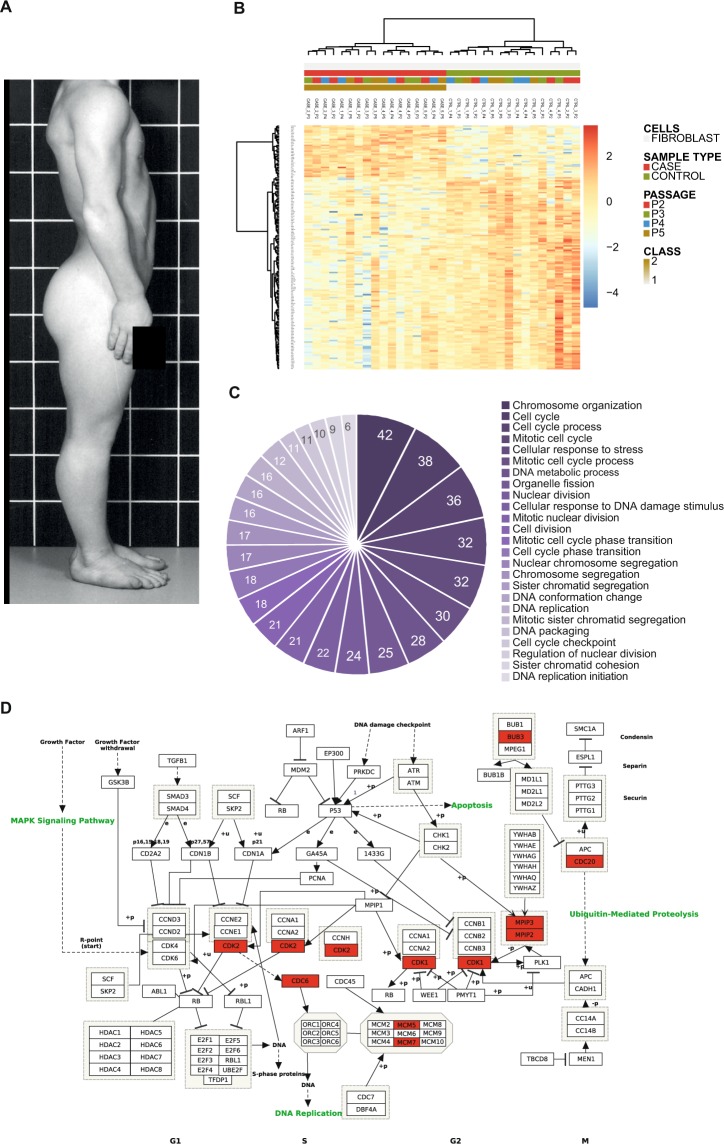
Transcriptome analysis of fibroblasts from CHH cases and healthy controls. (**A**) Male individual with CHH with disproportionate short stature and hair hypoplasia. (**B**) Heatmap showing genes that are significantly differentially expressed between fibroblasts from CHH cases and healthy controls as well as hierarchical clustering of the samples based on the expression of all these genes. Higher expression is marked in orange/red, lower in blue. The gene names (on the left) together with sample codes (above each column) can be visualized in the high-resolution image. An identical list of the genes included in the heatmap, in identical order, together with additional information is available also as Supplementary Table S4. Numbering of patient samples is identical to the numbering in Supplementary Table S1. Cases/controls and passages 1/2/3/4/5 are color-coded. For each of the five cases and five controls, we analyzed 2–4 samples from different passages. (**C**) Pie chart showing the 25 most enriched Gene Ontology, Biological processes terms (GO_BP categories) in CHH fibroblasts, based on genes that are significantly downregulated in the STRT analysis. Numbers depict the number of genes involved in each category. (**D**) KEGG Pathway analysis of the genes that are significantly downregulated in CHH fibroblasts (marked in red). Images were obtained by KEGG, Kanehisa Laboratories.

Five adults with CHH, all homozygous for the previously described g.70 A > G *RMRP* mutation^2^, donated a skin biopsy for fibroblast cultures. All had typical phenotypic manifestations of CHH (see Supplementary Table S1, Fig. 1A). Five age- and sex-matched healthy adults volunteered for skin biopsies as controls.

### Cell growth is abnormal in CHH fibroblasts

Despite identical procedures when culturing fibroblasts from skin biopsies, we observed clear differences in cell growth between cases and controls. In primary cultures of skin biopsies, the CHH subjects’ cells required a median of 33 days to progress from passage 1 to passage 4, while the control fibroblasts required a median of 23 days. This difference in growth rate persisted when frozen fibroblasts were recultured (see Supplementary Fig. S1). After culturing an equal number of fibroblasts and measuring cell counts daily for the following three days, the cases’ cell numbers were lower already at day 1 as compared to control cells (see Supplementary Fig. S1). The number of case fibroblasts in daily measurements was 67–21% of the control cell counts throughout the culture period.

### RNA sequencing reveals 35 Up- and 130 downregulated genes in CHH fibroblasts

The quality of RNA samples, extracted from passage 2–5 fibroblasts from 5 CHH cases (in total 20 samples) and 5 matched controls (19 samples), was high and similar in case and control samples (see Supplementary Table S2). 10 ng of total RNA from each sample was analyzed by the modified single-cell tagged reverse transcription (STRT) sequencing method targeting 5′ ends of transcripts and transcription start sites^15,16^. Four samples (two case passages and two control passages) were excluded due to low 5′ capture rate, indicative of partial RNA degradation, leaving 18 and 17 acceptable samples for analysis, respectively (see Supplementary Table S3). The qualified samples averaged 3.17 million unique sequence reads per sample; of these 2.84 million were successfully mapped to the reference genome (hg19), and 1.99 million of these mapped to the 5′ coding part of known genes. These numbers did not differ between the case and control groups of samples (see Supplementary Table S3). The sequencing data have been deposited at the European Nucleotide Archive (https://www.ebi.ac.uk/ena) under the accession number PRJEB23608.

The passage of cells did not have a major effect on the transcriptome as shown by hierarchical clustering (see Fig. 1B) and principal component analysis (see Supplementary Fig. S2) of the case and control samples. Comparing cases and controls irrespective of passage, there were 35 genes that were significantly upregulated, and 130 genes significantly downregulated in CHH fibroblasts (see Supplementary Table S4).

Quantitative real-time reverse transcriptase PCR (qRT-PCR) validation of *CDK2*, *IFITM1*, *CDKN1A* and *BCL2L1* gene expression was in line with the results of the STRT RNA-seq data (see Supplementary Fig. S3). The expression of *CDK2* (by the independent samples *t*-test p-value 0.0087), *IFITM1* (p-value 0.0203), *CDKN1* (p-value 0.0113) and *BCL2L1* (p-value 0.0255) differed significantly between the CHH patients and controls. Significantly between the CHH patients and controls.

### Downregulated genes are connected to the cell cycle pathways

The genes that were downregulated in CHH cases compared to controls were significantly connected to the cell cycle (p-value < 5.1E-4) (see Fig. 1C). The downregulated Gene Ontology (GO) Biological processes categories involving chromosome organization, mitotic cell cycle, and cell cycle were strongly enriched in this group of genes. Correspondingly, the GO Cellular component and molecular function categories involving chromosomes and DNA packaging and binding were also enriched in the downregulated genes. Looking at enriched Kyoto Encyclopedia of Genes and Genomes (KEGG) pathways in the downregulated genes (see Fig. 1D, Supplementary Table S5A), one finds a corresponding enrichment of DNA replication and cell cycle pathways^17–19^.

### Upregulated genes involve PI3K-Akt signaling pathway

The GO Biological process “Regulation of molecular function” was enriched in the genes that were upregulated in CHH case fibroblasts as compared with controls, and Cellular Component GO categories involving e.g., extracellular exosomes, vesicles and organelles were significantly enriched in these cells. Furthermore, a set of genes involved in the Molecular function GO category enzyme activator activity was enriched in the CHH case fibroblasts. Finally, the KEGG pathway involving PI3K-Akt signaling was significantly upregulated (p-value < 2.2E-3) in the CHH case cells (see Supplementary Fig. S4, Supplementary Table S5B).

### CHH fibroblasts demonstrate prolonged cell cycle due to the delay in progression from G2 to G1

**Figure 2:**
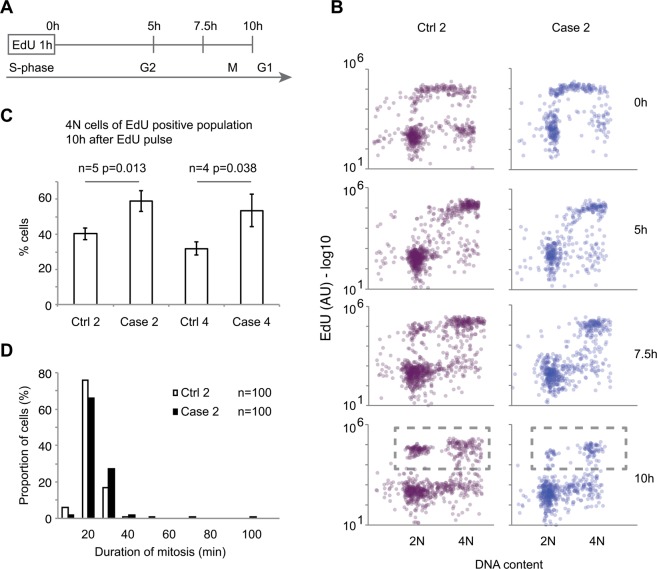
CHH fibroblasts are delayed in G2 phase. (**A**) Schematic presentation of the setup used in B and C. Fibroblasts were pulse-labeled with EdU and samples were harvested at the indicated time points. (**B**) CHH fibroblasts are delayed in transition from G2 to G1. The indicated cell lines were followed as outlined in A. Each circle corresponds to one fibroblast. (**C**) CHH fibroblasts are delayed in transition from G2 to G1. Quantification of fibroblasts denoted in rectangle in B, showing percentage of 4 N cells of EdU positive population 10 h after EdU pulse. Students t-test. Error bars show s.e.m. (**D**) The graph shows quantification of mitotic duration for the indicated cell lines. There was no major mitotic delay in CHH fibroblasts.

To directly assess cell cycle progression, we followed CHH fibroblasts by pulse-labeling and time-lapse microscopy. The absolute majority of both CHH and control cells remained viable, as 5 out of 800 control cells and 2 out of 800 CHH cells died when monitored by time-lapse microscopy for 29 h. Further, no difference in the number of dead cells was detected by visual inspection during cell culturing. This indicates that reduced accumulation of CHH case cells was not due to excessive cell death. Similarly, we found no difference in the proportion of CHH and control cells that incorporated EdU during a 30 h exposure, suggesting that reduced proliferation of CHH cells was due to a prolonged cell cycle rather than to cell cycle exit (see Supplementary Fig. S5). We therefore pulse-labeled S-phase cells with EdU and followed the DNA content of labeled cells using high content microscopy (see Fig. 2A). Five hours after an EdU pulse, the majority of both control and CHH cells had reached 4N DNA content, indicating that S-phase duration was similar between the cell types (see Fig. 2B). During the subsequent five hours, cells started passing mitosis, as evidenced by the rise of an EdU-positive population with 2N DNA content. Interestingly, we found that 10 h after an EdU pulse, CHH cells showed a higher proportion of 4N DNA content compared to control cells (see Figs 2B,C and S6), indicating that CHH cells were delayed in the progression from G2 to G1.

### The duration of mitosis is normal in CHH Fibroblasts

To assess if the delay occurred in G2-phase or in mitosis, we measured the duration of mitosis by time-lapse microscopy. In both cell types, more than 90% of cells completed mitosis within 30 min. Although we cannot exclude that CHH cells showed a minor delay in mitosis, only 3% of cells required more than 40 minutes to complete mitosis (see Fig. 2D). This suggested that the cell cycle delay observed in Fig. 2C was not due to a mitotic delay, but rather that CHH cells were delayed specifically in the passage from G2 phase to mitosis.

## Discussion

We demonstrated slow cell growth, defective cell cycle progression, and transcriptome differences in fibroblasts from patients with CHH compared to healthy controls. Impaired growth of cultured CHH fibroblasts was consistent with previous reports^9^ and was observed in primary fibroblast cultures from skin biopsies, as well as after reculturing of frozen cells.

The CHH gene was originally positionally cloned as the first disease-causing lncRNA gene^2^. The causative role of the Finnish mutation has been established by genetic studies earlier^20–22^, and therefore the study of the functional effects of *RMRP* was based on samples from patients carrying the Finnish founder mutation. As the causative mutation is non-coding and regulatory, and all overexpression models likely very unphysiological, we did not attempt such experiments to replenish the fibroblasts with an external *RMRP* gene construct.

Our findings are in good agreement with the clinical manifestations of CHH. When looking at individual up- and downregulated genes, more than half could be assigned to cellular functions that are likely to be important for clinical manifestations of CHH (see Supplementary Table S6). Only two genes overlapped with previously reported up- or downregulated genes in CHH^23^. This discrepancy is likely due to the use of different cell types (peripheral blood lymphocytes vs. fibroblasts) and different methodology, possibly with higher sensitivity of our assays.

We performed whole-transcriptome analysis of fibroblasts from both CHH cases and controls and found approximately three times more significantly downregulated than upregulated genes in CHH cases. The results were independent of the passage, suggesting that the difference was not influenced by progressive defects caused by cell culture conditions. Further analysis of GO category and KEGG pathway enrichment within downregulated genes revealed a clear signature of perturbations in several components of the cell cycle and cell cycle progression, consistent with earlier studies^23^. Although we did not perform a rescue experiment with wild-type *RMRP*, we used *RMRP*-mutation negative fibroblasts as control cells.

Our novel finding of the possibly impaired PI3K-Akt signaling in CHH fibroblasts deserves further studies. Enrichment of the KEGG pathway involving PI3K-Akt signaling within the upregulated genes in case cells is in agreement with the CHH phenotype, as the pathway has been shown to be involved in growth plate chondrocyte differentiation and regulation of endochondral bone growth in mice^24^. The pathway also plays a role in several malignancies, including lymphomas^25^, and may contribute to the high risk of non-Hodgkin lymphomas in patients with CHH^26^. These observations may have therapeutic implications and should be explored in future studies. All these findings led us to study in more detail which specific components of the cell cycle mechanism were defective in CHH.

To further validate the biological effects of the transcriptome profile changes and to gain further insight into slower cell growth in patient fibroblasts, we opted for direct observation of cell cycle effects. We applied pulse-labelling and live cell recordings to assess cell cycle progress in single cells. The results showed unequivocally that cell death or exiting from cell cycle did not account for the overall slowed proliferation effects in CHH fibroblasts. Furthermore, the S phase progressed at an identical pace in both case and control fibroblasts. Instead, case cells accumulated at the G2 phase, suggesting a defect in progression to mitosis, which took a similar time for both case and control cells to complete.

**Figure 3:**
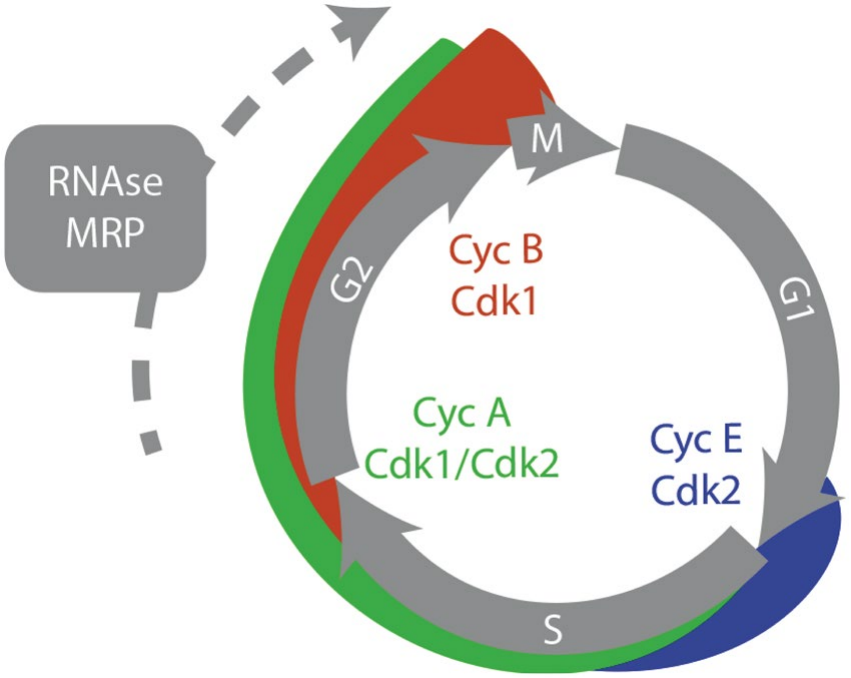
Schematic of key CDK complexes and *RMRP* function during the cell cycle. CHH fibroblasts delay in G2 phase. Although effects are pleiotropic and affect many cell cycle regulators, the main Cyclin-CDK complexes driving progression through G2 phase show differential regulation in CHH cells. First, mRNA levels of CDK2 that stimulates Cyclin A/B-CDK1 activation at the S/G2 border are reduced. Second, CHH cells show reduced mRNA levels of direct and indirect regulators of CDK1 activity, as Cdc25C and MAST-L.

Taken together, our findings from fibroblast gene expression patterns and cell cycle measurements on single fibroblasts, consistently indicate that *RMRP* mutations in CHH patients interfere with the cell cycle through multiple target genes. Our results strongly support the hypothesis that *RMRP* as a lncRNA has pleiotropic effects on a major part of the cell cycle network rather than acting predominantly through a few target genes. This fits well with the broad and even changes on multiple targets in the transcriptomic assay. However, the broad changes through the cell cycle are orchestrated by a limited set of actors, most notably Cyclin-dependent kinases (CDKs). CDK2 drives progression through S-phase and helps activating CDK1 at the S/G2 border, which results in build-up of CDK1 activity that eventually triggers mitotic entry (see Fig. 3)^27,28^. We noted that in addition to regulators of CDK1 target phosphorylation such as CDC25C and MAST-L, CDK2 mRNA levels are lower in CHH patient cells, whereas mRNA for the potent CDK inhibitor CDKN1A is upregulated. Our observation of a G2 delay in CHH cells is thus consistent with reduced activity and levels of the main players promoting progression through G2 phase (see Fig. 3).

The functions of lncRNAs are for the most part unknown. Broad regulatory effects on cell cycle have been documented on some lncRNAs, such as for LINC00152 regulating progression through mitosis^29^ and for GAS5 regulating G0/G1 arrest and apoptosis^30^. Most functional lncRNA studies are aimed at understanding the role of lncRNAs in cancer progression and metastasis and are based on cancer cell models. The effects of individual lncRNAs may thus be difficult to identify among the multiple alterations inherent in malignant cells. Functional studies on naturally occurring mutations in nonmalignant cells are rare and may have the potential of dissecting individual gene effects in more physiological cell environments. Our findings on cell cycle defects, particularly G2 progression to mitosis, in fibroblasts from CHH cases with known CHH-causing mutations in *RMRP*, thus add to our limited understanding on the functions of lncRNAs.

## Methods

### Ethics statement

The study protocol was approved by the Institutional Research Ethics Committee of the Helsinki University Hospital and University of Helsinki, Finland (76/13/03/03/2011) in accordance with the Helsinki Declaration. All study participants were adult and signed an informed consent at recruitment. One participant gave a written informed consent for publication of identifying information/images in an online open-access publication.

### Study participants

Skin punch biopsies from the left forearm were obtained for fibroblast cultures from five patients with CHH and from five healthy controls, all ethnic Finns. All patient-control pairs were matched for sex and age, aiming at an age difference of not more than five years. Patients were clinically assessed, and peripheral blood DNA was Sanger sequenced for *RMRP* to confirm the genotype (data not shown)^31^.

### Fibroblast cultures

The skin biopsy samples were put in sterile fibroblast culture media; Dulbecco’s Modified Eagle Medium with 4.5 g/L glucose (DMEM; BioWhittaker, Lonza, Verviers, Belgium), 20% Fetal Bovine Serum (FBS Premium; Biowest SAS, Nuaille, France), 50 IU/ml penicillin and 50 µg/ml streptomycin (Gibco, Life Technologies Europe, NN Bleiswijk, Netherlands). The skin biopsies were cut into small fragments in Phosphate-Buffered Saline (PBS; Gibco, Life Technologies, Carlsbad, CA) on a Petri dish and transferred to Falcon tubes containing 3 ml PBS (w/o Mg^2+^ and Ca^2+^) and 1,000 U/ml Collagenase Type II: Clostridium histolyticum (Gibco). The tubes were incubated at 37 °C for 2 h. Collagenase inactivation was performed by addition of 3 ml ice cold medium. The tubes were mixed by vortexing and tissue lysates were centrifuged for 10 min, 150 × *g* at 4 °C. The supernatants were removed, the cell pellets were re-suspended in 900 µl media, and transferred to T-25 flasks (Corning Life Sciences, New York, NY). 4 ml of media were added to each flask and they were incubated in a 5% CO_2_ humidified incubator at 37 °C for 1–2 weeks. Fibroblast culture media was changed every 3–4 days. Once the wells became confluent, the cells were detached using 0.25% trypsin/2.21 mM EDTA (Corning Life Sciences, New York, NY) and passed into larger flasks. After 3^rd^ passage the percentage of FBS in media was reduced to 15%. The cells were subcultured at regular intervals up to passage six in order to keep adherent cells healthy and actively growing. To compare cell growth in cases *vs* controls, the same number of cells (1.5 × 10^5^) was cultured in the 6-well plates (Nunc AS, Thermo Fisher Scientific, Roskilde, Denmark) (three wells for cases and three for controls) and the cells were counted at 24, 48 and 72 h.

### RNA extraction

RNA from the fibroblasts was isolated using the RNAeasy Mini kit (Qiagen, Hilden, Germany) according to the manufacturer’s instructions. RNA integrity was assessed on an Agilent 2100 Bioanalyzer (Agilent Technologies, Santa Clara, CA) at the Biomedicum Functional Genomics Unit (FuGU, Helsinki, Finland). RNA concentrations were measured using a Qubit 2.0 Fluorometer (Thermo Fisher, Carlsbad, CA).

### RNA sequencing

RNA transcriptome analysis was performed using a modified version of the STRT protocol^32^. 10 ng of total RNA were reversely transcribed to cDNA and amplified to make an Illumina-compatible sequencing library. Artificial RNA molecules (ERCC RNA Spike-In Mix 1; Life Technologies) were added, enabling normalisation of expression data^32^. In total, the library was amplified through 25 PCR cycles: unique molecular identifiers (UMIs) were used to reduce amplification biases^33^.

The library was sequenced on three lanes of Illumina HiSeq2000 (Illumina, San Diego, CA) by using single-end 59 bp reads at the Bioinformatics and Expression Analysis (BEA) core facility at Karolinska Institutet, Huddinge, Sweden. Sequence data was converted to fastq files using Casava 1.8.2 (Illumina). Demultiplexing by the barcodes, exclusion of redundant reads by the UMIs, quality-based filtering, trimming, alignment, quantitation and normalization of expression levels, statistical tests on differential expression, illustration of the expression levels by heatmap and quality control were performed using the STRTprep pipeline available at https://github.com/shka/STRTprep/tree/v3dev ^32^. Differentially expressed genes between cases and controls were those with fluctuation p-value < 0.05 (as an assurance of degree of the variation, like as fold-change based thresholding) and with differential-expression q-values < 0.05 (as an assurance of difference between the groups; estimated by SAMstrt^16^ from the pipeline), as described in Supplementary data in Krjutskov *et al*.^32^.

### qRT-PCR validation

Six genes, with robust expression and significantly different expression levels in CHH patients and healthy controls by STRT RNA-seq were selected for technical validation by qRT-PCR analysis. We included three CHH patients and controls in the qRT-PCR validation and used the same RNA that was used for the initial whole-transcriptome analysis. cDNA was transcribed from 1 μg of total RNA using the QuantiTect Reverse Transcription Kit cDNA synthesis kit (Qiagen) according to the manufacturer’s protocol. qRT-PCR assays were performed in quintuplicates using the CXF96 Real-Time system (Bio-Rad Laboratories, Herkules, CA) and using the TaqMan *CDK2*, *IFITM1*, *CDKN1A*, *BCL2L1*, *ACTB* and *TBP* gene expression assays Hs01548894_m1, Hs00705137_s1, Hs00355782_m1, Hs00236329_m1, Hs99999903_m1 and Hs00427620_m1, respectively (Applied Biosystems, Thermo Fisher Scientific, Carlsbad, CA). *ACTB* and *TBP* were used as reference genes for data normalization. Threshold cycle (Ct) values were determined using CFX Manager Software (Bio-Rad Laboratories). Relative expression was calculated using the comparative Ct or 2ΔΔCt method^34,35^.

### Sequencing data GO/KEGG analysis

The DAVID database (v6.8 at https://david.ncifcrf.gov/home.jsp), was used to identify enriched GO categories (GO BP_FAT, GO MF_FAT and GO_CC_FAT) and KEGG pathways within the set of differentially expressed genes.

### Determination of cell cycle delay by EdU incorporation

To label the cells in S phase, cells in passage 4 or 5 were plated at a density of 3,000 cells per well in a 96-well imaging plate (BD Falcon, Franklin Lakes, NJ) and cultured for one day prior to pulse with 1 μM 5-ethynyl-2′-deoxyuridine (EdU; Molecular Probes, Eugene, OR) for 1 h. The cells were fixed using 3.7% formaldehyde (Sigma Aldrich, St Louis, MO) for 5 min and permeabilised in −20 °C methanol (Sigma Aldrich) for 2 min followed by incubation with DAPI. For the click-chemistry reaction, cells were incubated in a solution containing 100 mM Tris (pH 6.8), 1 mM CuS0_4_, 100 mM ascorbic acid and azid fluorophore (#A10277, Invitrogen, Carlsbad, CA) for 1 h at room temperature. Images were acquired on an ImageXpress imaging system (Molecular Devices, Sunnyvale, CA) using a Nikon Plan Fluor ELWD 20 × 0.45 NA objective, resulting in 640 × 479 pixel 16-bit images. Image analysis was performed using CellProfiler^36^. Background was subtracted using the CellProfiler inbuilt illumination function with 120-pixel block size. Nuclei were segmented based on DAPI intensity using a manually-set threshold and the integrated intensity of DAPI and EdU fluorescence was measured.

### Determination of duration of mitosis by time-lapse video recording

After 1 day, cells were seeded as described above, the plate was placed on the stage of a Leica DMI6000 Imaging system and maintained at 37 °C with 5% CO_2_ supply. Differential interference contrast time-lapse video recordings were made at 10-min intervals for 29 h using a 20×, 0.4 NA objective. Duration of mitosis was defined as time from cell rounding to anaphase.

## Supplementary information

Supplementary materials

## Data Availability

All data generated or analysed during this study are included in this published article (and its Supplementary Information files).
