# Supplementary material for: The human long non-coding RNA gene RMRP has pleiotropic effects and regulates cell-cycle progression at G2
Source: Sci Rep. 2019 Sep 24;9:13758. doi: 10.1038/s41598-019-50334-6 (PMC6760211; doi:10.1038/s41598-019-50334-6)
Supplement: Supplementary file 1 — Supplementary materials [file 41598_2019_50334_MOESM1_ESM.pdf]

The human long non-coding RNA gene *RMRP* has pleiotropic effects and regulates cell-cycle progression at G2

Svetlana Vakkilainen[1, 2¶*]

Tiina Skoog[3¶]

Elisabet Einarsdottir [2, 3, 4]

Anna Middleton[5]

Minna Pekkinen[1, 2]

Tiina Öhman[6]

Shintaro Katayama[3]

Kaarel Krjutškov [3, 4, 7]

Panu E. Kovanen[8]

Markku Varjosalo[6]

Arne Lindqvist[6]

Juha Kere[2, 3, 4, 9]

Outi Mäkitie[1, 2, 10]

1) Children's Hospital, University of Helsinki and Helsinki University Hospital, Helsinki, 00290, Finland

2) Folkhälsan Research Center, Institute of Genetics, Helsinki, 00290, Finland

3) Department of Biosciences and Nutrition, Karolinska Institutet, Huddinge, SE-141 83, Sweden

4) Molecular Neurology Research Program, University of Helsinki, Helsinki, 00290, Finland

5) Department of Cell and Molecular Biology, Karolinska Institutet, Stockholm, SE-171 77, Sweden

6) Institute of Biotechnology, and Helsinki Institute of Life Science, University of Helsinki, Helsinki, 00290, Finland

7) Competence Centre on Health Technologies, Tartu, 50410, Estonia

8) Department of Pathology, University of Helsinki, and HUSLAB, Helsinki University Hospital, Helsinki, 00290, Finland

9) Department of Medical and Molecular Genetics, King's College, London, WC2R 2LS, UK

10) Department of Molecular Medicine and Surgery, Karolinska Institutet and Clinical Genetics, Karolinska University Hospital, Stockholm, SE-171 77, Sweden

*Corresponding author:

E-mail: svetlana.kostjukovits@helsinki.fi, Tel. +358-9-191 25453, Fax. +358-9-191 25073.

¶These authors contributed equally to this work.

Supplementary Figure S1. The impaired growth of cultured fibroblasts from cases with CHH compared with fibroblasts from healthy controls. Equal amounts of fibroblasts (1.5×10^5 cells per well) from three subjects with CHH and three healthy individuals were cultured for 72 h. Cells were counted every 24 h. Images demonstrate differences in fibroblast densities between cases and controls at 24, 48 and 72 hours of culturing.

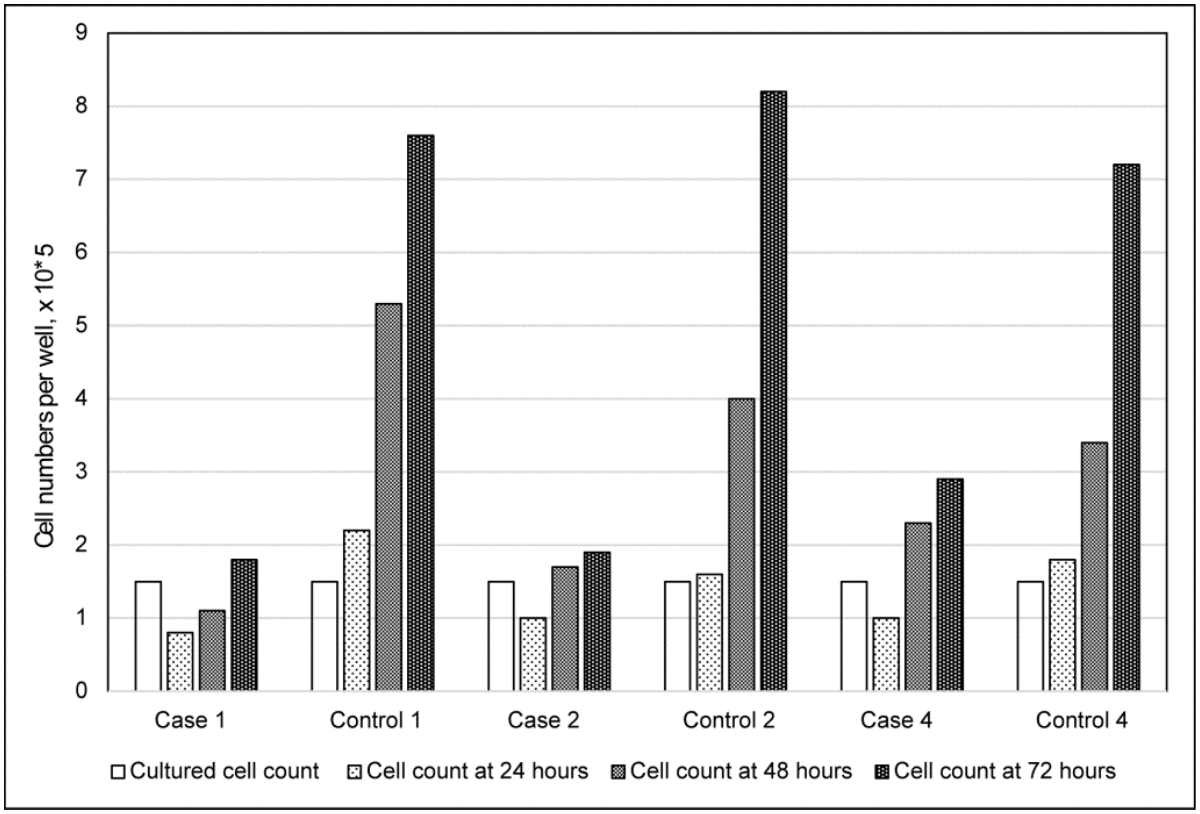

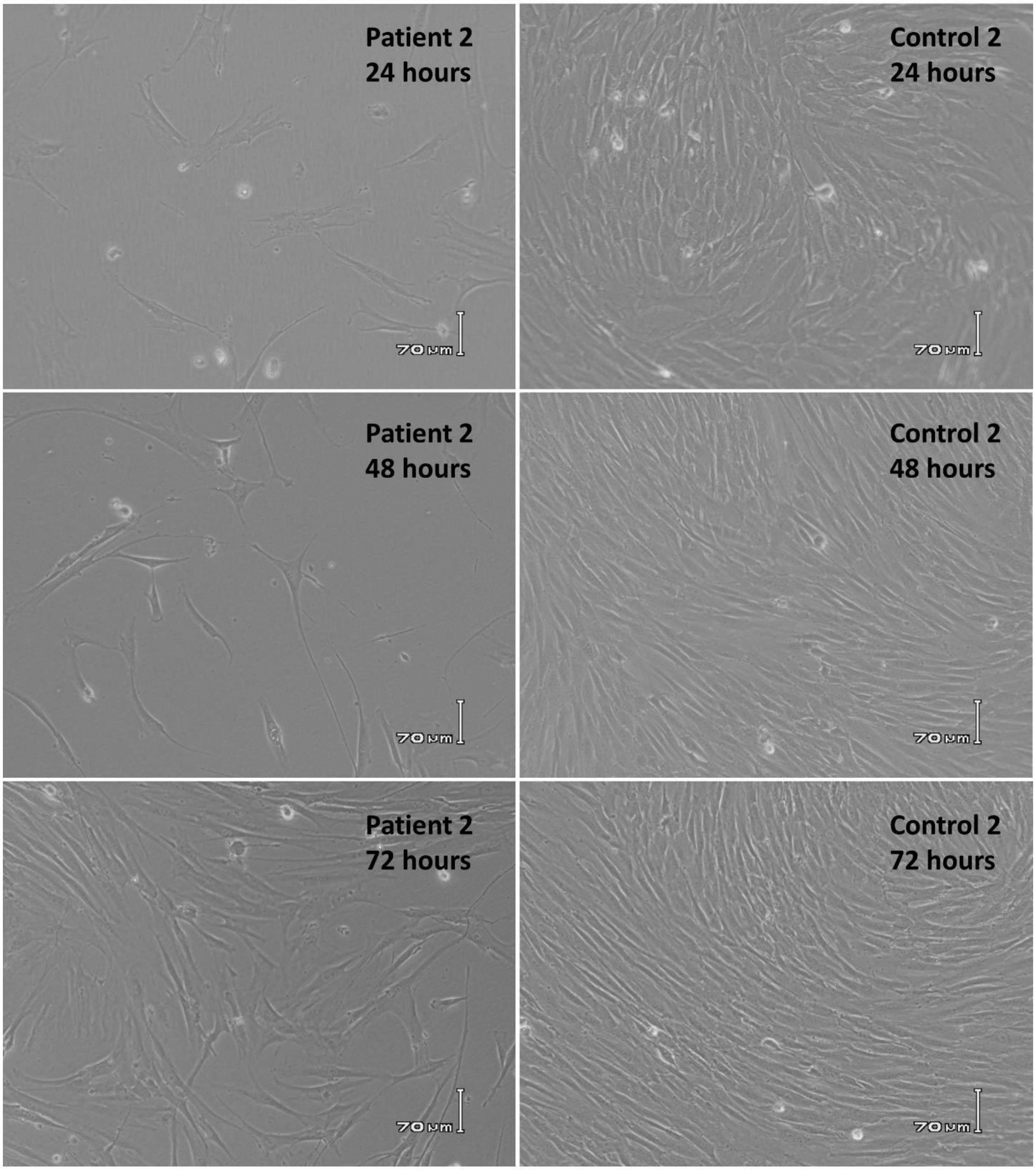
Patient 2
24 hours
70 µm
Control 2
24 hours
70 µm
Patient 2
48 hours
70 µm
Control 2
48 hours
70 µm
Patient 2
72 hours
70 µm
Control 2
72 hours
70 µm

Supplementary Table S1. Characteristics of the five Finnish cases with CHH who participated in the study.

**Case #**	**Sex**	**Age group (yrs)**	**Skin biopsy used for**	***RMRP* genotype, all homozygous**	**Height (cm)**	**Immunological and other features**	**Hb, g/L***	**TLC, cells x 10^9/L***
1	M	40-50	FC	g.70A>G	138.5	Bronchiectasis, CRS	150	3.08
2	M	60-65	FC, CCA	g.70A>G	129.0	BCC	148	**0.92**
3	F	60-65	FC	g.70A>G	120.0	BCC, CRS, severe varicella	136	**1.01**
4	M	20-30	FC, CCA	g.70A>G	129.0	Chronic otitis media, CRS, IVIG, Hirschsprung disease	129	**0.60**
5	F	30-40	FC	g.70A>G	140.5	Asymptomatic	126	1.43

BCC basal cell carcinoma, CCA, cell cycle analysis, CRS chronic rhinosinusitis, F female, FC fibroblast culture, Hb hemoglobin, IVIG intravenous immunoglobulin replacement therapy, M male, *RMRP* RNA component of mitochondrial RNA processing endoribonuclease, TLC total lymphocyte count.

* Local laboratory reference values were applied. Numbers in bold indicate subnormal values for age.

Supplementary Figure S2. Principal component analysis of fibroblasts from cases and controls. Principal component analysis of fibroblasts from patients with CHH (red color) and controls (blue color). Different shapes depict fibroblast passages: ring for the second, triangle for the third, plus for the fourth and x for the fifth passage.

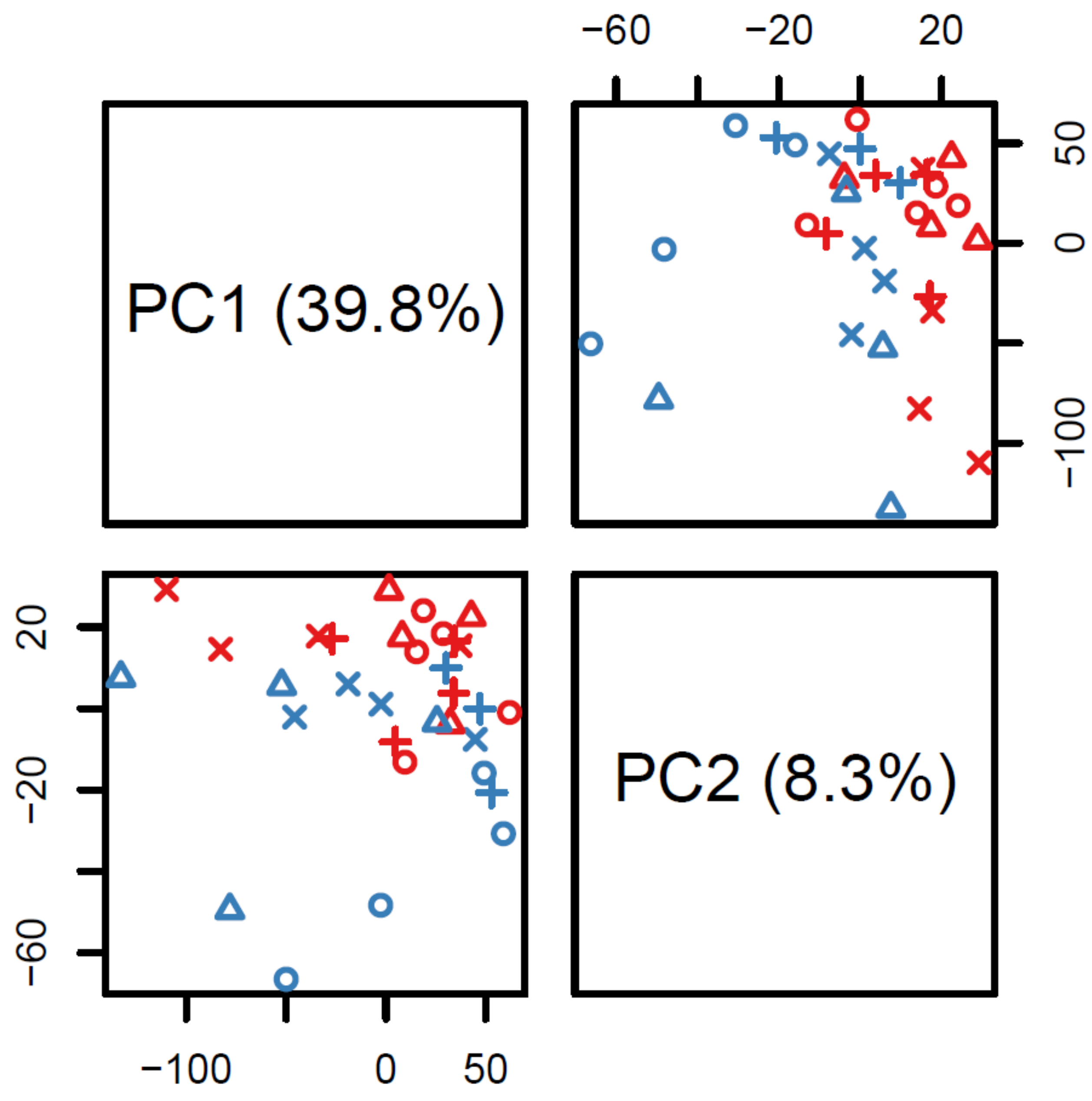

Supplementary Table S2. The quality of RNA in the five case and five control samples used for the STRT transcriptome analysis.

**Sample type**	**Passage**	**No. of samples**	**A_{260}/A_{280}**	**RIN**
Case	P2	5	2.01 (1.93-2.05)	10.0 (10.0-10.0)
Case	P3	5	2.10 (2.02-2.34)	9.9 (9.5-10.0)
Case	P4	5	2.12 (2.02-2.35)	10.0 (10.0-10.0)
Case	P5	5	2.06 (2.02-2.20)	10.0 (10.0-10.0)
**Case**	**All**	**20**	**2.07 (1.93-2.35)**	**10.0 (9.5-10.0)**
Control	P2	4	2.04 (2.02-2.06)	9.8 (9.3-10.0)
Control	P3	5	2.03 (2.02-2.06)	9.4 (8.7-10.0)
Control	P4	5	2.05 (2.03-2.07)	10.0 (9.8-10.0)
Control	P5	5	2.03 (2.01-2.05)	9.9 (9.6-10.0)
**Control**	**All**	**19**	**2.04 (2.01-2.07)**	**9.8 (8.7-10.0)**

Supplemental Figure S3. Validation of selected differentially expressed genes in CHH patients and healthy controls. Significant relative expression difference both with qRT-PCR and STRT methods were seen in *CDK2*, *IFITM1*, *CDKN1* and *BCL2L1* genes (p-value <0.05).

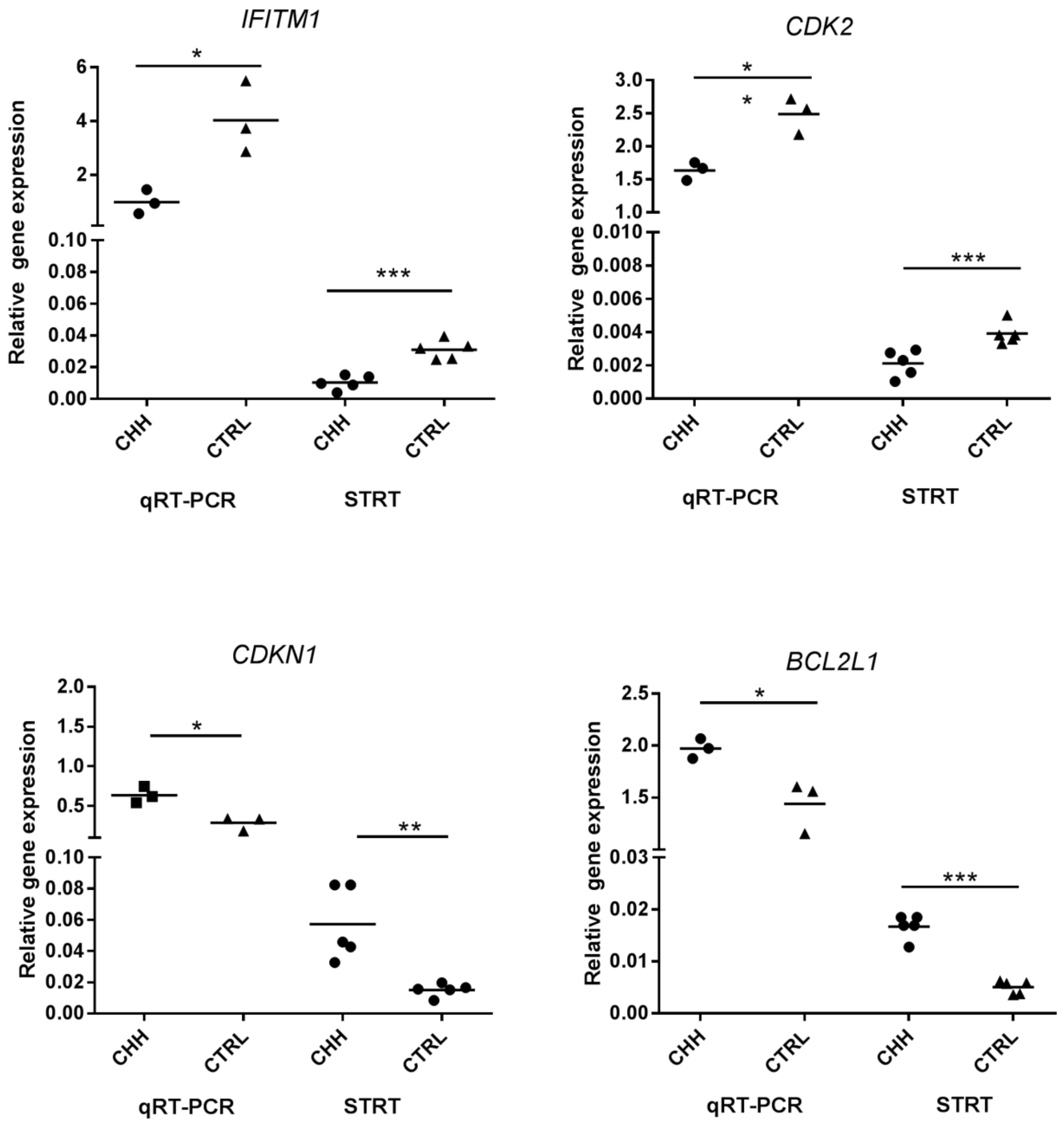

Supplementary Table S3. Raw data and QC of all CHH case and healthy control samples.

LIBRARY	WELL	NAME	PASSAGE	CLASS.TFE	SAMPLE TYPE	FORCE_ APPROVAL	CLASS.0	BLOCK.0	SPIKEIN_READS. OUTLIER	MAPPED/SPIKEIN. OUTLIER	SPIKEIN_5END_ RATE.OUTLIER	CODING_5END_ RATE.OUTLIER
RHH2	A1	CTRL_2_P3	P3	FIBROBLAST	CONTROL	FALSE	1	1	FALSE	FALSE	FALSE	FALSE
RHH2	A2	CTRL_3_P3	P3	FIBROBLAST	CONTROL	FALSE	1	1	FALSE	FALSE	FALSE	FALSE
RHH2	A3	CTRL_4_P3	P3	FIBROBLAST	CONTROL	FALSE	1	1	FALSE	FALSE	FALSE	FALSE
RHH2	A4	NA	P2	FIBROBLAST	CONTROL	FALSE	NA	NA	FALSE	FALSE	FALSE	FALSE
RHH2	A5	NA	P2	FIBROBLAST	CASE	FALSE	NA	NA	FALSE	FALSE	FALSE	FALSE
RHH2	A6	CASE_1_P5	P5	FIBROBLAST	CASE	FALSE	2	1	FALSE	FALSE	FALSE	FALSE
RHH2	A7	CASE_3_P5	P5	FIBROBLAST	CASE	FALSE	2	1	FALSE	FALSE	FALSE	FALSE
RHH2	A8	CASE_4_P5	P5	FIBROBLAST	CASE	FALSE	2	1	FALSE	FALSE	FALSE	FALSE
RHH2	B1	CASE_5_P5	P5	FIBROBLAST	CASE	FALSE	2	1	FALSE	FALSE	FALSE	FALSE
RHH2	B2	CTRL_1_P5	P5	FIBROBLAST	CONTROL	FALSE	1	1	FALSE	FALSE	FALSE	FALSE
RHH2	B3	CTRL_2_P4	P4	FIBROBLAST	CONTROL	FALSE	1	1	FALSE	FALSE	FALSE	TRUE
RHH2	B4	CTRL_3_P5	P5	FIBROBLAST	CONTROL	FALSE	1	1	FALSE	FALSE	FALSE	FALSE
RHH2	B5	CTRL_4_P5	P5	FIBROBLAST	CONTROL	FALSE	1	1	FALSE	FALSE	FALSE	FALSE
RHH2	B6	CTRL_5_P5	P5	FIBROBLAST	CONTROL	FALSE	1	1	FALSE	FALSE	FALSE	FALSE
RHH2	B7	CASE_1_P2	P2	FIBROBLAST	CASE	FALSE	2	1	FALSE	FALSE	FALSE	FALSE
RHH2	B8	CASE_2_P2	P2	FIBROBLAST	CASE	FALSE	2	1	FALSE	FALSE	FALSE	FALSE
RHH2	C1	CASE_3_P2	P2	FIBROBLAST	CASE	TRUE	2	1	TRUE	FALSE	FALSE	FALSE
RHH2	C2	CASE_4_P2	P2	FIBROBLAST	CASE	FALSE	2	1	FALSE	FALSE	FALSE	FALSE
RHH2	C3	CASE_5_P2	P2	FIBROBLAST	CASE	FALSE	2	1	FALSE	FALSE	FALSE	FALSE
RHH2	C4	CTRL_1_P2	P2	FIBROBLAST	CONTROL	FALSE	1	1	FALSE	FALSE	FALSE	FALSE
RHH2	C5	CTRL_2_P2	P2	FIBROBLAST	CONTROL	FALSE	1	1	FALSE	FALSE	FALSE	FALSE
RHH2	C6	CTRL_3_P2	P2	FIBROBLAST	CONTROL	FALSE	1	1	FALSE	FALSE	FALSE	FALSE
RHH2	C7	CTRL_4_P2	P2	FIBROBLAST	CONTROL	FALSE	1	1	FALSE	FALSE	FALSE	FALSE
RHH2	C8	CASE_2_P3	P3	FIBROBLAST	CASE	FALSE	2	1	FALSE	FALSE	FALSE	FALSE
RHH2	D1	CASE_4_P4	P4	FIBROBLAST	CASE	TRUE	2	1	TRUE	FALSE	FALSE	FALSE

RHH2	D2	CASE_1_P3	P3	FIBROBLAST	CASE	FALSE	2	1	FALSE	FALSE	FALSE	TRUE
RHH2	D3	CASE_3_P3	P3	FIBROBLAST	CASE	TRUE	2	1	TRUE	FALSE	FALSE	FALSE
RHH2	D4	CASE_4_P3	P3	FIBROBLAST	CASE	FALSE	2	1	FALSE	FALSE	FALSE	FALSE
RHH2	D5	CASE_5_P3	P3	FIBROBLAST	CASE	FALSE	2	1	FALSE	FALSE	FALSE	FALSE
RHH2	D6	CTRL_1_P3	P3	FIBROBLAST	CONTROL	FALSE	1	1	FALSE	FALSE	FALSE	FALSE
RHH2	D7	CTRL_5_P3	P3	FIBROBLAST	CONTROL	FALSE	1	1	FALSE	FALSE	TRUE	FALSE
RHH2	D8	CASE_1_P4	P4	FIBROBLAST	CASE	FALSE	2	1	FALSE	FALSE	FALSE	FALSE
RHH2	E1	CASE_2_P4	P4	FIBROBLAST	CASE	FALSE	2	1	FALSE	FALSE	FALSE	FALSE
RHH2	E2	CASE_3_P4	P4	FIBROBLAST	CASE	FALSE	2	1	FALSE	FALSE	FALSE	TRUE
RHH2	E3	CASE_5_P4	P4	FIBROBLAST	CASE	FALSE	2	1	FALSE	FALSE	FALSE	FALSE
RHH2	E4	CTRL_1_P4	P4	FIBROBLAST	CONTROL	FALSE	1	1	FALSE	FALSE	FALSE	FALSE
RHH2	E5	CTRL_3_P4	P4	FIBROBLAST	CONTROL	TRUE	1	1	TRUE	FALSE	FALSE	FALSE
RHH2	E6	NA	NA	FIBROBLAST	NA	FALSE	NA	NA	FALSE	FALSE	FALSE	FALSE
RHH2	E7	CTRL_4_P4	P4	FIBROBLAST	CONTROL	FALSE	1	1	FALSE	FALSE	FALSE	FALSE
RHH2	E8	CTRL_5_P4	P4	FIBROBLAST	CONTROL	FALSE	1	1	FALSE	FALSE	FALSE	FALSE
RHH2	F1	NA	NA	FIBROBLAST	NA	FALSE	NA	NA	FALSE	FALSE	FALSE	FALSE
RHH2	F2	NA	NA	FIBROBLAST	NA	FALSE	NA	NA	FALSE	FALSE	FALSE	FALSE
RHH2	F3	NA	NA	FIBROBLAST	NA	FALSE	NA	NA	FALSE	FALSE	FALSE	FALSE
RHH2	F4	NA	NA	FIBROBLAST	NA	FALSE	NA	NA	FALSE	FALSE	FALSE	FALSE
RHH2	F5	NA	NA	FIBROBLAST	NA	FALSE	NA	NA	FALSE	FALSE	FALSE	FALSE
RHH2	F6	NA	NA	FIBROBLAST	NA	FALSE	NA	NA	FALSE	FALSE	FALSE	FALSE
RHH2	F7	NA	NA	FIBROBLAST	NA	FALSE	NA	NA	FALSE	FALSE	FALSE	FALSE
RHH2	F8	NA	NA	FIBROBLAST	NA	FALSE	NA	NA	FALSE	FALSE	FALSE	FALSE

Supplementary Figure S4. P13K-AKT signaling pathway. Modified from http://www.genome.jp/kegg-bin/show_pathway?hsa04151

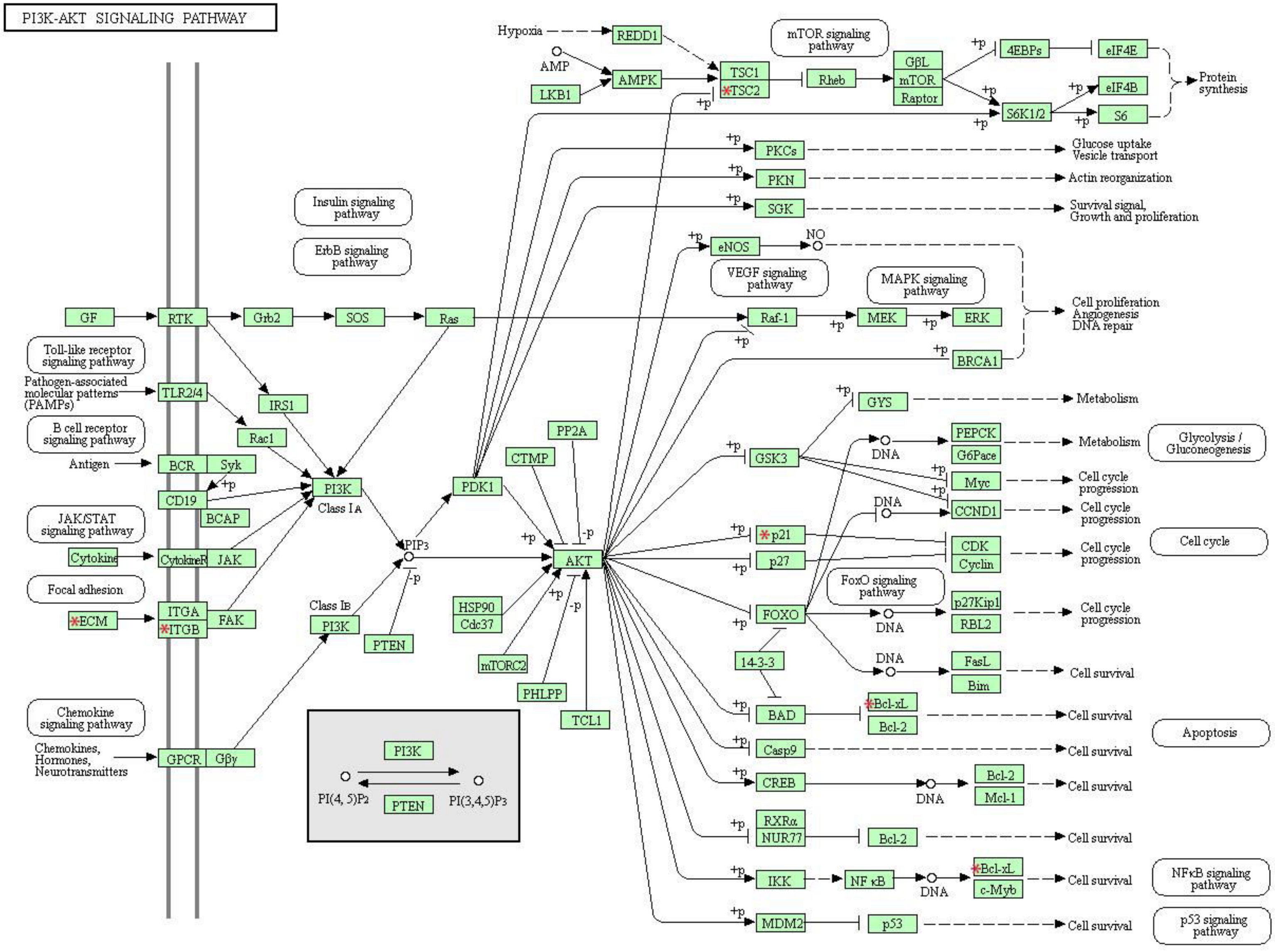

Supplementary Table S4. Significantly differentially expressed genes in fibroblasts from cases with CHH compared to fibroblasts from healthy controls.

	UPREGULATED IN CHH PATIENT FIBROBLASTS					DOWNREGULATED IN CHH PATIENT FIBROBLASTS					
Gene	diffexp Score.0	pvalue.0	qvalue.0	fluctuation.0	entrez_ geneID	Gene	diffexp Score.0	pvalue.0	qvalue.0	fluctuation.0	entrez_ geneID
STMN2	135,9	0,06872222	0	0	11075	PSG4	-135,95	0,06872222	0	5,9109E-153	5672
MGARP	108,4	0,06872222	0	9,44407E-35	84709	IFITM1	-127,5	0,06872222	0	3,44715E-93	8519
CLU	105,4	0,06872222	0	2,004E-235	1191	CGB5	-126,6	0,06872222	0	1,28777E-55	93659
PLXNB2	104,25	0,06872222	0	3,3348E-55	23654	RUNX3	-121,95	0,06872222	0	1,96001E-15	864
RGS16	102,75	0,06872222	0	3,24805E-26	6004	MTFP1	-111,9	0,06872222	0	1,45451E-16	51537
ADIRF	101,2	0,06872222	0	0	10974	HAPLN1	-109,75	0,06872222	0	2,9005E-116	1404
COMP	101,15	0,06872222	0	2,80158E-43	1311	PPP1R14A	-106,55	0,06872222	0	2,0631E-176	94274
GSTT1	98,7	0,07112821	0	6,96282E-60	2952	DEK	-105,9	0,06872222	0	1,65068E-35	7913
BCL2L1	98,2	0,07112821	0	7,02329E-57	598	XRRA1	-105,7	0,06872222	0	5,38423E-18	143570
HSPB7	96,4	0,07112821	0	0	27129	ACTG2	-101,7	0,06872222	0	9,2942E-301	72
WISP2	96,05	0,07112821	0	0,000423958	8839	GMFG	-100,9	0,06872222	0	0,003789175	9535
ZNF48	93,8	0,07112821	0,0118448	0,000455219	197407	G0S2	-95,7	0,07112821	0	2,71107E-06	50486
SPON2	93,4	0,07112821	0,0118448	2,13193E-67	10417	RHEBL1	-95	0,07112821	0	0,000834329	121268
MASP1	92,45	0,07112821	0,0118448	4,4638E-16	5648	H2AFZ	-94,6	0,07112821	0	9,2065E-142	3015
OSR1	91,95	0,07112821	0,0118448	7,7446E-49	130497	BIRC5	-93,2	0,07112821	0	2,48248E-35	332
VPS9D1	91,9	0,07112821	0,0118448	1,8573E-11	9605	POSTN	-93	0,07112821	0	1,04055E-72	10631
CEMIP	90,75	0,0727625	0,01864926	1,5242E-225	57214	ANKRD1	-92,65	0,07112821	0	0	27063
CDKN1A	88,85	0,07905102	0,01864926	5,8436E-123	1026	C19orf33	-92,1	0,07112821	0	4,7212E-194	64073
CLGN	88,85	0,07905102	0,01864926	0,001450054	1047	ASF1B	-91,75	0,07112821	0	1,64517E-26	55723
CLDN11	88,7	0,07905102	0,01864926	1,3021E-136	5010	LUM	-91,45	0,07112821	0	1,67617E-44	4060
KRTAP1-5	88,65	0,07905102	0,01864926	2,18806E-08	83895	SMC2	-91,25	0,07112821	0	3,3234E-18	10592
ADRA2A	88,05	0,07974528	0,01864926	3,0165E-06	150	DDX39A	-91,2	0,07112821	0	2,15689E-15	10212
CDC42EP2	86,95	0,08960909	0,03048749	3,41132E-11	10435	CRIP1	-90,3	0,07443023	0	8,1195E-220	1396
FN1	86,75	0,09018852	0,03048749	0	2335	PRIM1	-90,1	0,07443023	0	1,95436E-15	5557

SEPP1	85,8	0,09351613	0,03371213	8,81225E-13	864	RPL22L1	-90,05	0,07443023	0	8,3304E-122	200916
TSC2	85,55	0,09458594	0,03371213	6,19178E-13	7249	HLA-B	-89,85	0,07501136	0	7,8475E-237	3106
PROCR	85,25	0,09476866	0,03371213	5,80219E-07	10544	RRM2	-89,5	0,07666667	0	1,9932E-102	6241
HIST1H2BD	85	0,09547101	0,03371213	6,64273E-06	3017	MT1X	-88,3	0,07974528	0	4,7458E-240	4501
VAT1	83,8	0,09975	0,03703586	2,45696E-43	10493	PSAT1	-88,25	0,07974528	0	6,0491E-54	29968
LYNX1	83,7	0,09975	0,03703586	8,80603E-14	66004	POLE3	-88,05	0,07974528	0	4,96823E-12	54107
ARHGAP29	83,2	0,10141566	0,04063316	1,96517E-21	9411	C19orf48	-87,35	0,08663889	0	2,9219E-20	84798
UCHL1	82,35	0,1057033	0,04695618	2,0094E-99	7345	PRSS23	-86,65	0,09018852	0	6,47739E-80	11098
GLRX	82,3	0,1057033	0,04695618	2,43964E-32	2745	HIST1H2AH	-86,35	0,09018852	0	7,66573E-56	85235
A4GALT	82,15	0,10698913	0,04695618	7,1356E-30	53947	GRAMD3	-86,25	0,09018852	0	1,98068E-71	65983
CASKIN2	81,6	0,11359574	0,04952064	0,027819075	57513	CH25H	-86,2	0,09018852	0	1,80713E-17	9023
						DNAJC9	-86,2	0,09018852	0	4,99684E-70	23234
						HMGB3	-85,5	0,09458594	0	1,26963E-32	3149
						CCDC34	-85,15	0,09476866	0	1,60826E-14	91057
						CENPF	-84,9	0,09547101	0	9,7624E-109	1063
						TRIP13	-84,3	0,09926	0,01391294	2,05926E-13	9319
						GSTM3	-84,15	0,09926	0,01391294	9,18125E-35	2947
						HIST1H2AM	-84,1	0,09926	0,01391294	5,6404E-165	8336
						MCM5	-84,1	0,09926	0,01391294	2,14766E-07	4174
						MZT1	-84,05	0,09926	0,01391294	1,10265E-07	440145
						SUPT3H	-83,6	0,09975	0,01391294	0,003447133	8464
						RRP7A	-83,55	0,09975	0,01391294	1,1543E-115	27341
						SLC9A3R1	-83,45	0,09975	0,01391294	0,013925004	9368
						PCK2	-83,4	0,09975	0,01391294	2,04268E-27	5106
						SHCBP1	-83,1	0,10158333	0,01391294	0,000672083	79801
						ATAD2	-82,65	0,10530682	0,01391294	5,27342E-73	29028
						CDK2	-82,65	0,10530682	0,01391294	2,24192E-15	1017
						TMEM154	-82,6	0,10530682	0,01391294	0,000963418	201799

						MICA	-82,55	0,10530682	0,01391294	1,46112E-19	100507436
						MXD3	-82,3	0,1057033	0,01391294	8,18391E-08	83463
						CENPM	-81,8	0,11126882	0,01391294	2,4576E-20	79019
						CDCA8	-81,35	0,11558163	0,01391294	2,49452E-30	55143
						ASPM	-81,25	0,11558163	0,01391294	5,0634E-11	259266
						HIST2H2AC	-81,2	0,11558163	0,01391294	7,96706E-50	8338
						HAUS8	-80,95	0,1170049	0,01391294	5,10429E-12	93323
						HLA-C	-80,95	0,1170049	0,01391294	2,1178E-269	3107
						UBE2T	-80,55	0,1208381	0,02086941	1,2194E-31	29089
						RAD51AP1	-80,5	0,1208381	0,02086941	0,015320369	10635
						CKAP2L	-79,3	0,13509483	0,02086941	1,76048E-05	150468
						CDC20	-79,15	0,13509483	0,02086941	6,05988E-76	991
						CHN1	-79	0,13509483	0,02086941	3,89358E-06	1123
						HIST1H1A	-79	0,13509483	0,02086941	5,34108E-91	3024
						MLLT3	-78,65	0,13954237	0,02767943	0,001216973	4300
						KPNA2	-78,35	0,14339583	0,02767943	4,04505E-97	3838
						PDLIM1	-78,25	0,14382927	0,02767943	5,171E-63	9124
						GTSE1	-78,15	0,14382927	0,02767943	6,47139E-53	51512
						DIAPH3	-77,7	0,150796	0,02767943	0,000192071	81624
						KTN1	-77,35	0,15402308	0,02767943	1,21257E-13	3895
						TYMS	-77,35	0,15402308	0,02767943	2,48204E-07	7298
						FEN1	-77,25	0,15402308	0,02767943	1,17669E-19	2237
						PARP1	-76,8	0,15715	0,02767943	1,61848E-16	142
						UBE2C	-76,75	0,15715	0,02767943	3,4605E-150	11065
						IL1RL1	-76,65	0,15715	0,02767943	5,54436E-39	9173
						KIF4A	-76,65	0,15715	0,02767943	2,18335E-06	24137
						IFITM3	-76,6	0,15715	0,02767943	4,6283E-123	10410
						MTHFD2	-76,6	0,15715	0,02767943	5,2893E-108	10797

						NCAPH2	-76,6	0,15715	0,02767943	0,000130565	29781
						GSTM1	-76,4	0,15808392	0,02767943	4,68758E-97	2944
						TLE4	-76,25	0,15925517	0,03102709	3,28432E-15	7091
						UCP2	-76,15	0,16044863	0,03102709	1,1523E-24	7351
						RAD1	-75,45	0,16963158	0,03102709	9,53423E-06	5810
						TRAPPC4	-75,45	0,16963158	0,03102709	5,64912E-51	51399
						MKI67	-75,2	0,17212037	0,03102709	1,75468E-07	4288
						FUS	-75	0,17212037	0,03478235	4,21088E-63	2521
						EIF4EBP1	-74,95	0,17212037	0,03478235	5,03727E-89	1978
						DUT	-74,9	0,17212037	0,03478235	7,88368E-34	1854
						RFC3	-74,9	0,17212037	0,03478235	2,4584E-08	5983
						CALB2	-74,8	0,172375	0,03478235	2,4124E-125	794
						BLMH	-74,75	0,17241818	0,03478235	0,002650582	642
						LRRFIP1	-74,55	0,17321302	0,03478235	2,4174E-15	9208
						LSM2	-74,25	0,17637283	0,03478235	3,11147E-44	57819
						PBK	-73,95	0,18136389	0,03924696	3,04126E-41	55872
						NUSAP1	-73,9	0,18136389	0,03924696	5,37707E-38	51203
						ZWINT	-73,8	0,18136389	0,03924696	5,13157E-10	11130
						ULK4	-73,75	0,18136389	0,03924696	9,62575E-08	54986
						KRT14	-73,6	0,18214402	0,03924696	2,2637E-24	3861
						MASTL	-73,55	0,18214402	0,03924696	0,000416442	84930
						SMC4	-73,45	0,18268011	0,03924696	5,2651E-16	10051
						NANS	-73,25	0,18367105	0,03924696	8,22647E-14	54187
						TRIM59	-72,95	0,18742526	0,04290635	0,041913741	286827
						LPCAT2	-72,65	0,18932	0,04290635	2,31759E-08	54947
						SPARC	-72,6	0,1896194	0,04290635	2,6642E-262	6678
						CDC6	-72,35	0,19119417	0,04290635	2,84434E-17	990
						MYLK	-72,35	0,19119417	0,04290635	1,968E-106	4638

						HIST1H1B	-72	0,19744601	0,04290635	4,14777E-21	3009
						COL3A1	-71,85	0,19744601	0,04382577	8,63546E-38	1281
						MCM7	-71,75	0,19824651	0,04382577	2,98605E-36	4176
						CLSPN	-71,6	0,19933716	0,04382577	2,83669E-50	63967
						CXXC1	-71,6	0,19933716	0,04382577	0,000497492	30827
						IER3	-71,55	0,19981963	0,04382577	1,36E-53	8870
						KIF23	-71,45	0,20065611	0,04382577	4,3282E-05	9493
						GINS2	-71,4	0,20108559	0,04382577	3,38536E-23	51659
						FAM111B	-71,2	0,20433186	0,04382577	4,49966E-05	374393
						DDIT4	-71,15	0,20433186	0,04382577	1,70671E-25	54541
						PHGDH	-71,15	0,20433186	0,04382577	9,75747E-78	26227
						CDC25C	-70,75	0,21119565	0,04667833	5,67475E-05	995
						BUB3	-70,65	0,21227586	0,04667833	4,64564E-19	9184
						PGF	-70,65	0,21227586	0,04667833	1,56476E-34	5228
						HIST1H1E	-70,6	0,21277468	0,04667833	5,56949E-56	3008
						AURKB	-70,55	0,21325641	0,04667833	3,05009E-42	9212
						RMI1	-70,35	0,21582427	0,04980201	0,03246484	80010
						HIST1H3C	-70,3	0,21582427	0,04980201	1,58211E-16	8352
						HIST1H3B	-70,05	0,22004303	0,04980201	4,49169E-07	8358
						UNG	-70	0,22004303	0,04980201	7,49763E-11	7374
						EXOSC9	-69,85	0,22244715	0,04980201	1,59347E-16	5393
						MT1F	-69,85	0,22244715	0,04980201	2,76692E-11	4494

Supplementary Figure S5. EdU incorporation during 30 h exposure. Indicated fibroblast cell lines were incubated with EdU for 30h. Error bars show s.e.m.

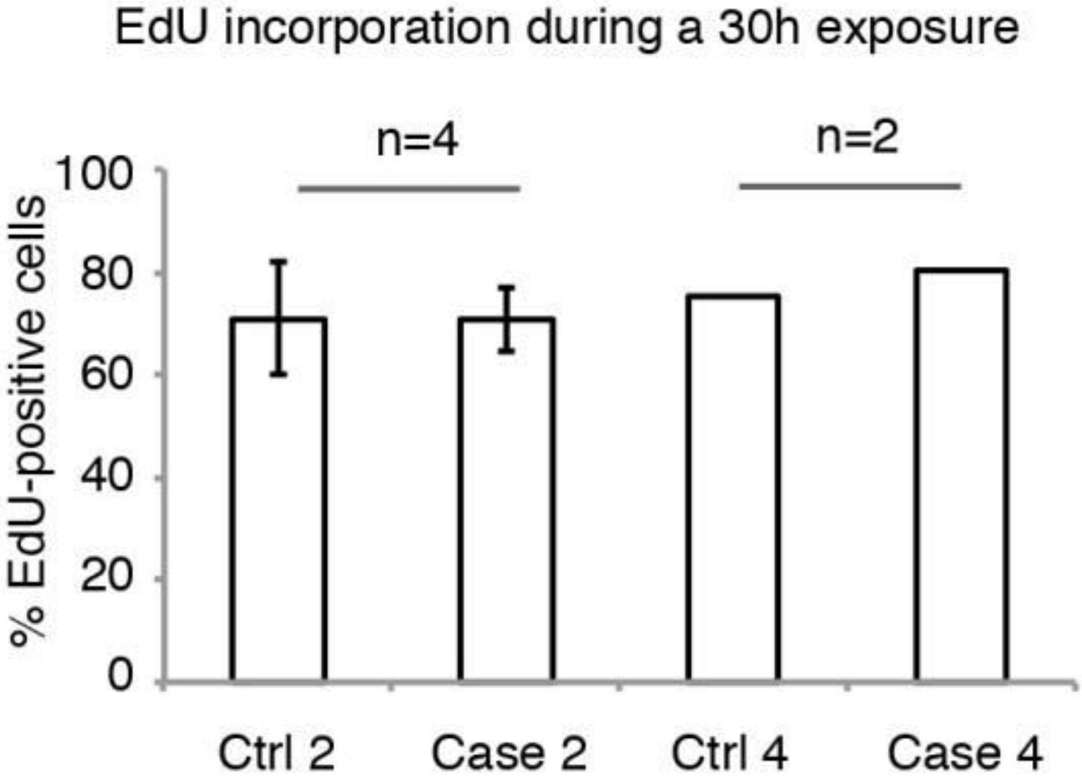

Supplementary Table S5. Enriched KEGG pathways* in significantly A.) downregulated and B.) upregulated genes in the case fibroblasts.

A.

**Enriched pathway, downregulated genes**	***P*-Value**	**Count**
hsa03030:DNA replication	0,0000099	6
hsa04110:Cell cycle	0,0005079	7
hsa03410:Base excision repair	0,0024364	4
hsa05322:Systemic lupus erythematosus	0,0047512	6
hsa00240:Pyrimidine metabolism	0,0103857	5
hsa05034:Alcoholism	0,0148958	6
hsa05202:Transcriptional misregulation in cancer	0,0491960	5
hsa00480:Glutathione metabolism	0,0659161	3

B.

**Enriched pathway, upregulated genes**	***P*-Value**	**Count**
hsa04151:PI3K-Akt signaling pathway	0,0021956	5

* KEGG copyright permission number 180245 [1].

1. Kanehisa, M. *et al*. KEGG: new perspectives on genomes, pathways, diseases and drugs. *Nucleic Acids Research* **45(D1)**, D353-D361. doi: 10.1093/nar/gkw1092 (2017).

Supplementary Table S6. Pathways that may be relevant for the features of CHH and that involve genes that were up- or downregulated in fibroblasts from cases with CHH compared with fibroblasts from healthy controls.

Features of cartilage-hair hypoplasia	Genes relevant to the particular feature (in alphabetic order)
Pathogenesis	
Ribosomal RNA cleavage	*RRP7A* [1]
Messenger RNA cleavage	*DDX39A*[2], *DEK*[3], *FUS*[4], *LSM2*[5]
Impaired cell cycle	*ASPM*[6], *AURKB*[7], *BUB3*[8], *CDC6*[9], *CDCA8*[10], *CDC20*[11], *CDC25C*[12], *CDK2*[13], **CDKN1A**[14], *CLSPN*[15], *EIF4EBP1*[16], *FEN1*[17], *KRT14*[18], *MASTL*[19], *MCM5*[20], *MCM7*[21], *MKI67*[22], *PBK*[23], **PLXNB2**[24], *POLE3*[25], *PSAT1*[26], *RRM2*[27], *SKAP2L*[28], *TRIP13*[29], **TSC2**[30], *UBE2C*[31]
Increased apoptosis	**BCL2L1**[32], *BIRC5*[33], **CDKN1A**[34], *CLSPN*[15], *DDIT4*[35], *G0S2*[36], *GINS2*[37], *GTSE1*[38], *IER3*[39], *MCM5*[40], *MTFP1*[41], *PARP1*[42], **SEPP1**[43], **TSC2**[44], *UBE2C*[45], *UCP2*[46],
Disrupted telomere machinery	*AURKB*[47], *DDX39A*[48], *FEN1*[49], *PARP1*[50], *UNG*[51]
Chondrodysplasia	
Bone and cartilage formation	*CDC6*[52], **COMP**[53], **FN1**[54], *HAPLN1*[55], *IFITM1*[56], *NANS*[57], **OSR1**[58], **PLXNB2**[59], *POSTN2*[60], *RUNX3*[61], *SLC9A3R1*[62], *SPARC*[63], **STMN2**[64]
Ligamental laxity	**CLDN11**[65], *COL3A1*[66], *LUM*[67]
Immunodeficiency	
Increased susceptibility to infections	*CH25H*[68], *EIF4EBP1*[69], *IER3*[70], *IFITM1*[71], *IFITM3*[72], *IL1RL1*[73], *LRRFIP1*[74], **MASP1**[75], *MICA*[76], **PROCR**[77], **SPON2**[78], *TRIM59*[79], *UNG*[80], **VPS9D1**[81]
Increased incidence of malignancies	*ATAD2*[82], *AURKB*[83], *BLMH*[84], *BUB3*[85], *CCDC34*[86], *CDC6*[87], *CDC25C*[12], *CDCA8*[88], **CEMIP**[89], **CLDN11**[90], **CLU**[91], *DEK*[92], *FEN1*[93], *G0S2*[94], *GINS2*[95], *GSTM1*[96], **GSTT1**[97], **HSPB7**[98], *IER3*[99], *IFITM1*[100], *KIF23*[101], *KPNA2*[102], *LPCAT2*[103], *LRRFIP1*[104], *MASTL*[105], *MCM7*[106], *MICA*[107], *MLLT3*[108], *MT1F*[109], *MTHFD2*[110], *MXD3*[111], *NUSAP1*[112], **OSR1**[113], *PARP1*[114], *PBK*[115], *PCK2*[116], *PDLIM1*[117], *PHGDH*[118], *POSTN2*[119], *PPP1R14A*[120], *PSAT1*[26], *RFC3*[121], *RRM2*[122], *RUNX3*[123], *SHCBP1*[124], *SPARC*[125], *TLE4*[126], *TOP2A*[127], *TRAPPC4*[128], *TRIM59*[129], *TRIP13*[130], **TSC2**[44], *UBE2C*[131], *UBE2T*[132], **UCHL1**[133], **VPS9D1**[134], **WISP2**[135]
Impaired hematopoiesis	
Anemia	*BIRC5*[136], *GMFG*[137], **GSTT1**[97], *MLLT3*[138]
Lymphocyte deficiency	*CXXC1*[139], *GMFG*[137], *HMGB3*[140], *NCAPH2*[141], *PARP1*[142], **PROCR**[143], *RUNX3*[144], *TLE4*[145], *UCP2*[146]
Hirschsprung disease (impaired neuronal migration)	*CALB2*[147], **PLXNB2**[148], **STMN2**[149]
Impaired spermatogenesis	*AURKB*[150], **BCL2L1**[151], *CDC25C*[152], **CLDN11**[153], **CLGN**[154], **OSR1**[155], *PBK*[156], **SEPP1**[157], **TSC2**[158], **UCHL1**[159]
Hair hypoplasia	**CLDN11**[160], **KRTAP1-5**[161], *TLE4*[162]

Genes in **bold** and *italics* were up- and down-regulated in fibroblasts of patients with cartilage-hair hypoplasia compared with healthy controls.

References for Supplementary Table 6.

1. Maserati, M., et al., *Identification of four genes required for mammalian blastocyst formation.* Zygote, 2014. **22**(3): p. 331-9.
2. Sugiura, T., K. Sakurai, and Y. Nagano, *Intracellular characterization of DDX39, a novel growth-associated RNA helicase.* Exp Cell Res, 2007. **313**(4): p. 782-90.

3. Soares, L.M., et al., *Intron removal requires proofreading of U2AF/3' splice site recognition by DEK.* Science, 2006. **312**(5782): p. 1961-5.
4. Masuda, A., et al., *Position-specific binding of FUS to nascent RNA regulates mRNA length.* Genes Dev, 2015. **29**(10): p. 1045-57.
5. Ingelfinger, D., et al., *The human LSm1-7 proteins colocalize with the mRNA-degrading enzymes Dcp1/2 and Xrnl in distinct cytoplasmic foci.* Rna, 2002. **8**(12): p. 1489-501.
6. Capecchi, M.R. and A. Pozner, *ASPM regulates symmetric stem cell division by tuning Cyclin E ubiquitination.* Nat Commun, 2015. **6**: p. 8763.
7. Shin, J., et al., *Aurkb/PP1-mediated resetting of Oct4 during the cell cycle determines the identity of embryonic stem cells.* Elife, 2016. **5**: p. e10877.
8. Kalitsis, P., et al., *Bub3 gene disruption in mice reveals essential mitotic spindle checkpoint function during early embryogenesis.* Genes Dev, 2000. **14**(18): p. 2277-82.
9. Borlado, L.R. and J. Mendez, *CDC6: from DNA replication to cell cycle checkpoints and oncogenesis.* Carcinogenesis, 2008. **29**(2): p. 237-43.
10. Yamanaka, Y., et al., *Loss of Borealin/DasraB leads to defective cell proliferation, p53 accumulation and early embryonic lethality.* Mech Dev, 2008. **125**(5-6): p. 441-50.
11. Chen, Z., et al., *Functional roles of PC-PLC and Cdc20 in the cell cycle, proliferation, and apoptosis.* Cell Biochem Funct, 2010. **28**(4): p. 249-57.
12. Boutros, R., V. Lobjois, and B. Ducommun, *CDC25 phosphatases in cancer cells: key players? Good targets?* Nat Rev Cancer, 2007. **7**(7): p. 495-507.
13. Kaldis, P. and E. Aleem, *Cell cycle sibling rivalry: Cdc2 vs. Cdk2.* Cell Cycle, 2005. **4**(11): p. 1491-4.
14. Di Giorgio, E., et al., *The control operated by the cell cycle machinery on MEF2 stability contributes to the downregulation of CDKN1A and entry into S phase.* Mol Cell Biol, 2015. **35**(9): p. 1633-47.
15. Petermann, E., T. Helleday, and K.W. Caldecott, *Claspin promotes normal replication fork rates in human cells.* Mol Biol Cell, 2008. **19**(6): p. 2373-8.
16. Dowling, R.J., et al., *mTORC1-mediated cell proliferation, but not cell growth, controlled by the 4E-BPs.* Science, 2010. **328**(5982): p. 1172-6.
17. Finger, L.D., et al., *The wonders of flap endonucleases: structure, function, mechanism and regulation.* Subcell Biochem, 2012. **62**: p. 301-26.
18. Alam, H., et al., *Novel function of keratins 5 and 14 in proliferation and differentiation of stratified epithelial cells.* Mol Biol Cell, 2011. **22**(21): p. 4068-78.
19. Diril, M.K., et al., *Loss of the Greatwall Kinase Weakens the Spindle Assembly Checkpoint.* PLoS Genet, 2016. **12**(9): p. e1006310.
20. Tsuruga, H., et al., *Expression, nuclear localization and interactions of human MCM/P1 proteins.* Biochem Biophys Res Commun, 1997. **236**(1): p. 118-25.
21. Zhang, X.Y., et al., *Interaction of MCM7 and RACK1 for activation of MCM7 and cell growth.* Am J Pathol, 2013. **182**(3): p. 796-805.
22. Hou, Y.Y., et al., *MicroRNA-519d targets MKi67 and suppresses cell growth in the hepatocellular carcinoma cell line QGY-7703.* Cancer Lett, 2011. **307**(2): p. 182-90.
23. Rizkallah, R., et al., *Identification of the oncogenic kinase TOPK/PBK as a master mitotic regulator of C2H2 zinc finger proteins.* Oncotarget, 2015. **6**(3): p. 1446-61.
24. Xia, J., et al., *Semaphorin-Plexin Signaling Controls Mitotic Spindle Orientation during Epithelial Morphogenesis and Repair.* Dev Cell, 2015. **33**(3): p. 299-313.
25. Bolognese, F., et al., *The Pole3 bidirectional unit is regulated by MYC and E2Fs.* Gene, 2006. **366**(1): p. 109-16.
26. Yang, Y., et al., *PSAT1 regulates cyclin D1 degradation and sustains proliferation of non-small cell lung cancer cells.* Int J Cancer, 2015. **136**(4): p. E39-50.

27. Kittler, R., et al., *An endoribonuclease-prepared siRNA screen in human cells identifies genes essential for cell division.* Nature, 2004. **432**(7020): p. 1036-40.
28. Yumoto, T., et al., *Radmis, a novel mitotic spindle protein that functions in cell division of neural progenitors.* PLoS One, 2013. **8**(11): p. e79895.
29. Ma, H.T. and R.Y. Poon, *TRIP13 Regulates Both the Activation and Inactivation of the Spindle-Assembly Checkpoint.* Cell Rep, 2016. **14**(5): p. 1086-99.
30. Tapon, N., et al., *The Drosophila tuberous sclerosis complex gene homologs restrict cell growth and cell proliferation.* Cell, 2001. **105**(3): p. 345-55.
31. Ben-Eliezer, I., et al., *Appropriate expression of Ube2C and Ube2S controls the progression of the first meiotic division.* Faseb j, 2015. **29**(11): p. 4670-81.
32. Clausen, L.N., et al., *Genetic variants in the apoptosis gene BCL2L1 improve response to interferon-based treatment of hepatitis C virus genotype 3 infection.* Int J Mol Sci, 2015. **16**(2): p. 3213-25.
33. Lamers, F., et al., *Knockdown of survivin (BIRC5) causes apoptosis in neuroblastoma via mitotic catastrophe.* Endocr Relat Cancer, 2011. **18**(6): p. 657-68.
34. Chu, K., et al., *Computerized video time lapse study of cell cycle delay and arrest, mitotic catastrophe, apoptosis and clonogenic survival in irradiated 14-3-3sigma and CDKN1A (p21) knockout cell lines.* Radiat Res, 2004. **162**(3): p. 270-86.
35. Wolff, N.C., R.M. McKay, and J. Brugarolas, *REDD1/DDIT4-independent mTORC1 inhibition and apoptosis by glucocorticoids in thymocytes.* Mol Cancer Res, 2014. **12**(6): p. 867-77.
36. Wang, Y., et al., *Lipolytic inhibitor G0/G1 switch gene 2 inhibits reactive oxygen species production and apoptosis in endothelial cells.* Am J Physiol Cell Physiol, 2015. **308**(6): p. C496-504.
37. Zhang, X., et al., *Effect of GINS2 on proliferation and apoptosis in leukemic cell line.* Int J Med Sci, 2013. **10**(12): p. 1795-804.
38. Monte, M., et al., *The cell cycle-regulated protein human GTSE-1 controls DNA damage-induced apoptosis by affecting p53 function.* J Biol Chem, 2003. **278**(32): p. 30356-64.
39. Wu, M.X., et al., *IEX-1L, an apoptosis inhibitor involved in NF-kappaB-mediated cell survival.* Science, 1998. **281**(5379): p. 998-1001.
40. Ryu, S., et al., *Depletion of minichromosome maintenance protein 5 in the zebrafish retina causes cell-cycle defect and apoptosis.* Proc Natl Acad Sci U S A, 2005. **102**(51): p. 18467-72.
41. Tondera, D., et al., *Knockdown of MTP18, a novel phosphatidylinositol 3-kinase-dependent protein, affects mitochondrial morphology and induces apoptosis.* J Biol Chem, 2004. **279**(30): p. 31544-55.
42. Diamantopoulos, P.T., et al., *PARP1-driven apoptosis in chronic lymphocytic leukemia.* Biomed Res Int, 2014. **2014**: p. 106713.
43. Kabuyama, Y., et al., *Involvement of selenoprotein P in the regulation of redox balance and myofibroblast viability in idiopathic pulmonary fibrosis.* Genes Cells, 2007. **12**(11): p. 1235-44.
44. Inoki, K., T. Zhu, and K.L. Guan, *TSC2 mediates cellular energy response to control cell growth and survival.* Cell, 2003. **115**(5): p. 577-90.
45. Zhang, Z., et al., *Ubiquitin-conjugating enzyme E2C regulates apoptosis-dependent tumor progression of non-small cell lung cancer via ERK pathway.* Med Oncol, 2015. **32**(5): p. 149.
46. Qiao, C., et al., *UCP2-related mitochondrial pathway participates in oroxylin A-induced apoptosis in human colon cancer cells.* J Cell Physiol, 2015. **230**(5): p. 1054-63.
47. Mallm, J.P. and K. Rippe, *Aurora Kinase B Regulates Telomerase Activity via a Centromeric RNA in Stem Cells.* Cell Rep, 2015. **11**(10): p. 1667-78.
48. Yoo, H.H. and I.K. Chung, *Requirement of DDX39 DEAD box RNA helicase for genome integrity and telomere protection.* Aging Cell, 2011. **10**(4): p. 557-71.

49. Sampathi, S., et al., *Human flap endonuclease I is in complex with telomerase and is required for telomerase-mediated telomere maintenance.* J Biol Chem, 2009. **284**(6): p. 3682-90.
50. Aguennouz, M., et al., *Telomere shortening is associated to TRF1 and PARP1 overexpression in Duchenne muscular dystrophy.* Neurobiol Aging, 2011. **32**(12): p. 2190-7.
51. Vallabhaneni, H., et al., *Defective repair of uracil causes telomere defects in mouse hematopoietic cells.* J Biol Chem, 2015. **290**(9): p. 5502-11.
52. Bongers, E.M., et al., *Meier-Gorlin syndrome: report of eight additional cases and review.* Am J Med Genet, 2001. **102**(2): p. 115-24.
53. Motaung, S.C., P.E. Di Cesare, and A.H. Reddi, *Differential response of cartilage oligomeric matrix protein (COMP) to morphogens of bone morphogenetic protein/transforming growth factor-beta family in the surface, middle and deep zones of articular cartilage.* J Tissue Eng Regen Med, 2011. **5**(6): p. e87-96.
54. Dehne, T., et al., *Gene expression profiling of primary human articular chondrocytes in high-density micromasses reveals patterns of recovery, maintenance, re- and dedifferentiation.* Gene, 2010. **462**(1-2): p. 8-17.
55. Govindan, J. and M.K. Iovine, *Hapln1a is required for connexin43-dependent growth and patterning in the regenerating fin skeleton.* PLoS One, 2014. **9**(2): p. e88574.
56. Kim, B.S., et al., *IFITM1 increases osteogenesis through Runx2 in human alveolar-derived bone marrow stromal cells.* Bone, 2012. **51**(3): p. 506-14.
57. van Karnebeek, C.D., et al., *NANS-mediated synthesis of sialic acid is required for brain and skeletal development.* Nat Genet, 2016. **48**(7): p. 777-84.
58. Gao, Y., et al., *The zinc finger transcription factors Osr1 and Osr2 control synovial joint formation.* Dev Biol, 2011. **352**(1): p. 83-91.
59. Zhang, M., et al., *Microarray analysis of perichondral and reserve growth plate zones identifies differential gene expressions and signal pathways.* Bone, 2008. **43**(3): p. 511-20.
60. Zhang, F., et al., *Periostin: A Downstream Mediator of EphB4-Induced Osteogenic Differentiation of Human Bone Marrow-Derived Mesenchymal Stem Cells.* Stem Cells Int, 2016. **2016**: p. 7241829.
61. Wigner, N.A., et al., *Functional role of Runx3 in the regulation of aggrecan expression during cartilage development.* J Cell Physiol, 2013. **228**(11): p. 2232-42.
62. Schroeder, T.M., et al., *Gene profile analysis of osteoblast genes differentially regulated by histone deacetylase inhibitors.* BMC Genomics, 2007. **8**: p. 362.
63. Mendoza-Londono, R., et al., *Recessive osteogenesis imperfecta caused by missense mutations in SPARC.* Am J Hum Genet, 2015. **96**(6): p. 979-85.
64. Chiellini, C., et al., *Stathmin-like 2, a developmentally-associated neuronal marker, is expressed and modulated during osteogenesis of human mesenchymal stem cells.* Biochem Biophys Res Commun, 2008. **374**(1): p. 64-8.
65. Chiarelli, N., et al., *Transcriptome-Wide Expression Profiling in Skin Fibroblasts of Patients with Joint Hypermobility Syndrome/Ehlers-Danlos Syndrome Hypermobility Type.* PLoS One, 2016. **11**(8): p. e0161347.
66. Stembridge, N.S., et al., *Clinical, structural, biochemical and X-ray crystallographic correlates of pathogenicity for variants in the C-propeptide region of the COL3A1 gene.* Am J Med Genet A, 2015. **167a**(8): p. 1763-72.
67. Jepsen, K.J., et al., *A syndrome of joint laxity and impaired tendon integrity in lumican- and fibromodulin-deficient mice.* J Biol Chem, 2002. **277**(38): p. 35532-40.
68. Liu, S.Y., et al., *Interferon-inducible cholesterol-25-hydroxylase broadly inhibits viral entry by production of 25-hydroxycholesterol.* Immunity, 2013. **38**(1): p. 92-105.

69. Colina, R., et al., *Translational control of the innate immune response through IRF-7.* Nature, 2008. **452**(7185): p. 323-8.
70. Akilov, O.E., et al., *Enhanced susceptibility to Leishmania infection in resistant mice in the absence of immediate early response gene X-1.* J Immunol, 2009. **183**(12): p. 7994-8003.
71. Brass, A.L., et al., *The IFITM proteins mediate cellular resistance to influenza A H1N1 virus, West Nile virus, and dengue virus.* Cell, 2009. **139**(7): p. 1243-54.
72. Everitt, A.R., et al., *IFITM3 restricts the morbidity and mortality associated with influenza.* Nature, 2012. **484**(7395): p. 519-23.
73. Buckley, J.M., et al., *Increased susceptibility of ST2-deficient mice to polymicrobial sepsis is associated with an impaired bactericidal function.* J Immunol, 2011. **187**(8): p. 4293-9.
74. Bagashev, A., et al., *Leucine-rich repeat (in Flightless I) interacting protein-1 regulates a rapid type I interferon response.* J Interferon Cytokine Res, 2010. **30**(11): p. 843-52.
75. Sekine, H., et al., *The role of MASP-1/3 in complement activation.* Adv Exp Med Biol, 2013. **735**: p. 41-53.
76. Groh, V., et al., *Costimulation of CD8alphabeta T cells by NKG2D via engagement by MIC induced on virus-infected cells.* Nat Immunol, 2001. **2**(3): p. 255-60.
77. Kager, L.M., et al., *Overexpression of the endothelial protein C receptor is detrimental during pneumonia-derived gram-negative sepsis (Melioidosis).* PLoS Negl Trop Dis, 2013. **7**(7): p. e2306.
78. He, Y.W., et al., *The extracellular matrix protein mindin is a pattern-recognition molecule for microbial pathogens.* Nat Immunol, 2004. **5**(1): p. 88-97.
79. Kondo, T., M. Watanabe, and S. Hatakeyama, *TRIM59 interacts with ECSIT and negatively regulates NF-kappaB and IRF-3/7-mediated signal pathways.* Biochem Biophys Res Commun, 2012. **422**(3): p. 501-7.
80. Imai, K., et al., *Human uracil-DNA glycosylase deficiency associated with profoundly impaired immunoglobulin class-switch recombination.* Nat Immunol, 2003. **4**(10): p. 1023-8.
81. Tsalik, E.L., et al., *An integrated transcriptome and expressed variant analysis of sepsis survival and death.* Genome Med, 2014. **6**(11): p. 111.
82. Ciro, M., et al., *ATAD2 is a novel cofactor for MYC, overexpressed and amplified in aggressive tumors.* Cancer Res, 2009. **69**(21): p. 8491-8.
83. Wang, C., et al., *Aurora-B and HDAC synergistically regulate survival and proliferation of lymphoma cell via AKT, mTOR and Notch pathways.* Eur J Pharmacol, 2016. **779**: p. 1-7.
84. Okamura, Y., et al., *Identification of the bleomycin hydrolase gene as a methylated tumor suppressor gene in hepatocellular carcinoma using a novel triple-combination array method.* Cancer Lett, 2011. **312**(2): p. 150-7.
85. Morais da Silva, S., T. Moutinho-TRUEos, and C.E. Sunkel, *A tumor suppressor role of the Bub3 spindle checkpoint protein after apoptosis inhibition.* J Cell Biol, 2013. **201**(3): p. 385-93.
86. Gong, Y., et al., *CCDC34 is up-regulated in bladder cancer and regulates bladder cancer cell proliferation, apoptosis and migration.* Oncotarget, 2015. **6**(28): p. 25856-67.
87. Gonzalez, S., et al., *Oncogenic activity of Cdc6 through repression of the INK4/ARF locus.* Nature, 2006. **440**(7084): p. 702-6.
88. Hayama, S., et al., *Phosphorylation and activation of cell division cycle associated 8 by aurora kinase B plays a significant role in human lung carcinogenesis.* Cancer Res, 2007. **67**(9): p. 4113-22.
89. Zhang, D., et al., *Down-regulation of KIAA1199/CEMIP by miR-216a suppresses tumor invasion and metastasis in colorectal cancer.* Int J Cancer, 2017. **140**(10): p. 2298-2309.
90. Nissinen, L., et al., *Expression of claudin-11 by tumor cells in cutaneous squamous cell carcinoma is dependent on the activity of p38delta.* Exp Dermatol, 2016.

91. Zhang, H., et al., *Clusterin inhibits apoptosis by interacting with activated Bax.* Nat Cell Biol, 2005. **7**(9): p. 909-15.
92. Kavanaugh, G.M., et al., *The human DEK oncogene regulates DNA damage response signaling and repair.* Nucleic Acids Res, 2011. **39**(17): p. 7465-76.
93. Kucherlapati, M., et al., *Haploinsufficiency of Flap endonuclease (Fen1) leads to rapid tumor progression.* Proc Natl Acad Sci U S A, 2002. **99**(15): p. 9924-9.
94. Yim, C.Y., et al., *G0S2 Suppresses Oncogenic Transformation by Repressing a MYC-Regulated Transcriptional Program.* Cancer Res, 2016. **76**(5): p. 1204-13.
95. Rantala, J.K., et al., *Integrative functional genomics analysis of sustained polyploidy phenotypes in breast cancer cells identifies an oncogenic profile for GINS2.* Neoplasia, 2010. **12**(11): p. 877-88.
96. Zhong, S., et al., *Relationship between the GSTM1 genetic polymorphism and susceptibility to bladder, breast and colon cancer.* Carcinogenesis, 1993. **14**(9): p. 1821-4.
97. Lee, K.A., et al., *Increased frequencies of glutathione S-transferase (GSTM1 and GSTT1) gene deletions in Korean patients with acquired aplastic anemia.* Blood, 2001. **98**(12): p. 3483-5.
98. Lin, J., et al., *Downregulation of the tumor suppressor HSPB7, involved in the p53 pathway, in renal cell carcinoma by hypermethylation.* Int J Oncol, 2014. **44**(5): p. 1490-8.
99. Garcia, M.N., et al., *IER3 supports KRASG12D-dependent pancreatic cancer development by sustaining ERK1/2 phosphorylation.* J Clin Invest, 2014. **124**(11): p. 4709-22.
100. Hatano, H., et al., *IFN-induced transmembrane protein 1 promotes invasion at early stage of head and neck cancer progression.* Clin Cancer Res, 2008. **14**(19): p. 6097-105.
101. Takahashi, S., et al., *Downregulation of KIF23 suppresses glioma proliferation.* J Neurooncol, 2012. **106**(3): p. 519-29.
102. Yang, Y., et al., *Silencing of karyopherin alpha2 inhibits cell growth and survival in human hepatocellular carcinoma.* Oncotarget, 2017. **8**(22): p. 36289-36304.
103. Williams, K.A., et al., *A systems genetics approach identifies CXCL14, ITGAX, and LPCAT2 as novel aggressive prostate cancer susceptibility genes.* PLoS Genet, 2014. **10**(11): p. e1004809.
104. Ariake, K., et al., *GCF2/LRRFIP1 promotes colorectal cancer metastasis and liver invasion through integrin-dependent RhoA activation.* Cancer Lett, 2012. **325**(1): p. 99-107.
105. Vera, J., et al., *Greatwall promotes cell transformation by hyperactivating AKT in human malignancies.* Elife, 2015. **4**.
106. Ren, B., et al., *MCM7 amplification and overexpression are associated with prostate cancer progression.* Oncogene, 2006. **25**(7): p. 1090-8.
107. Groh, V., et al., *Tumour-derived soluble MIC ligands impair expression of NKG2D and T-cell activation.* Nature, 2002. **419**(6908): p. 734-8.
108. Krivtsov, A.V., et al., *Transformation from committed progenitor to leukaemia stem cell initiated by MLL-AF9.* Nature, 2006. **442**(7104): p. 818-22.
109. Yan, D.W., et al., *Downregulation of metallothionein 1F, a putative oncosuppressor, by loss of heterozygosity in colon cancer tissue.* Biochim Biophys Acta, 2012. **1822**(6): p. 918-26.
110. Tedeschi, P.M., et al., *Mitochondrial Methylenetetrahydrofolate Dehydrogenase (MTHFD2) Overexpression Is Associated with Tumor Cell Proliferation and Is a Novel Target for Drug Development.* Mol Cancer Res, 2015. **13**(10): p. 1361-6.
111. Barisone, G.A., et al., *Loss of MXD3 induces apoptosis of Reh human precursor B acute lymphoblastic leukemia cells.* Blood Cells Mol Dis, 2015. **54**(4): p. 329-35.
112. Fang, L., et al., *Downregulation of nucleolar and spindle-associated protein 1 expression suppresses cell migration, proliferation and invasion in renal cell carcinoma.* Oncol Rep, 2016. **36**(3): p. 1506-16.

113. Otani, K., et al., *Odd-skipped related 1 is a novel tumour suppressor gene and a potential prognostic biomarker in gastric cancer.* J Pathol, 2014. **234**(3): p. 302-15.
114. Fong, P.C., et al., *Inhibition of poly(ADP-ribose) polymerase in tumors from BRCA mutation carriers.* N Engl J Med, 2009. **361**(2): p. 123-34.
115. Nandi, A.K., et al., *Attenuation of DNA damage checkpoint by PBK, a novel mitotic kinase, involves protein-protein interaction with tumor suppressor p53.* Biochem Biophys Res Commun, 2007. **358**(1): p. 181-8.
116. Mendez-Lucas, A., et al., *Mitochondrial phosphoenolpyruvate carboxykinase (PEPCK-M) is a pro-survival, endoplasmic reticulum (ER) stress response gene involved in tumor cell adaptation to nutrient availability.* J Biol Chem, 2014. **289**(32): p. 22090-102.
117. Chen, H.N., et al., *PDLIM1 Stabilizes the E-Cadherin/beta-Catenin Complex to Prevent Epithelial-Mesenchymal Transition and Metastatic Potential of Colorectal Cancer Cells.* Cancer Res, 2016. **76**(5): p. 1122-34.
118. Possemato, R., et al., *Functional genomics reveal that the serine synthesis pathway is essential in breast cancer.* Nature, 2011. **476**(7360): p. 346-50.
119. Malanchi, I., et al., *Interactions between cancer stem cells and their niche govern metastatic colonization.* Nature, 2011. **481**(7379): p. 85-9.
120. Jin, H., et al., *Tumorigenic transformation by CPI-17 through inhibition of a merlin phosphatase.* Nature, 2006. **442**(7102): p. 576-9.
121. He, Z.Y., et al., *Up-Regulation of RFC3 Promotes Triple Negative Breast Cancer Metastasis and is Associated With Poor Prognosis Via EMT.* Transl Oncol, 2017. **10**(1): p. 1-9.
122. Duxbury, M.S. and E.E. Whang, *RRM2 induces NF-kappaB-dependent MMP-9 activation and enhances cellular invasiveness.* Biochem Biophys Res Commun, 2007. **354**(1): p. 190-6.
123. He, L., et al., *RUNX3 mediates suppression of tumor growth and metastasis of human CCRCC by regulating cyclin related proteins and TIMP-1.* PLoS One, 2012. **7**(3): p. e32961.
124. Feng, W., et al., *SHCBP1 is over-expressed in breast cancer and is important in the proliferation and apoptosis of the human malignant breast cancer cell line.* Gene, 2016. **587**(1): p. 91-7.
125. Bhoopathi, P., et al., *SPARC mediates Src-induced disruption of actin cytoskeleton via inactivation of small GTPases Rho-Rac-Cdc42.* Cell Signal, 2011. **23**(12): p. 1978-87.
126. Shin, T.H., et al., *TLE4 regulation of wnt-mediated inflammation underlies its role as a tumor suppressor in myeloid leukemia.* Leuk Res, 2016. **48**: p. 46-56.
127. Chen, T., et al., *Topoisomerase IIalpha in chromosome instability and personalized cancer therapy.* Oncogene, 2015. **34**(31): p. 4019-31.
128. Weng, Y.R., et al., *The role of ERK2 in colorectal carcinogenesis is partly regulated by TRAPPC4.* Mol Carcinog, 2014. **53 Suppl 1**: p. E72-84.
129. Lin, W.Y., et al., *Knockdown of tripartite motif 59 (TRIM59) inhibits tumor growth in prostate cancer.* Eur Rev Med Pharmacol Sci, 2016. **20**(23): p. 4864-4873.
130. Zhou, K., et al., *Loss of thyroid hormone receptor interactor 13 inhibits cell proliferation and survival in human chronic lymphocytic leukemia.* Oncotarget, 2017. **8**(15): p. 25469-25481.
131. Jiang, L., et al., *Knockdown of ubiquitin-conjugating enzyme E2C/UbcH10 expression by RNA interference inhibits glioma cell proliferation and enhances cell apoptosis in vitro.* J Cancer Res Clin Oncol, 2010. **136**(2): p. 211-7.
132. Wen, M., et al., *Elevated expression of UBE2T exhibits oncogenic properties in human prostate cancer.* Oncotarget, 2015. **6**(28): p. 25226-39.
133. Sanchez-Diaz, P.C., et al., *Ubiquitin carboxyl-terminal esterase L1 (UCHL1) is associated with stem-like cancer cell functions in pediatric high-grade glioma.* PLoS One, 2017. **12**(5): p. e0176879.

134. Yang, L., et al., *Dysregulation of long non-coding RNA profiles in human colorectal cancer and its association with overall survival.* Oncol Lett, 2016. **12**(5): p. 4068-4074.
135. Haque, I., et al., *CCN5/WISP-2 promotes growth arrest of triple-negative breast cancer cells through accumulation and trafficking of p27(Kip1) via Skp2 and FOXO3a regulation.* Oncogene, 2015. **34**(24): p. 3152-63.
136. Gurbuxani, S., et al., *Differential requirements for survivin in hematopoietic cell development.* Proc Natl Acad Sci U S A, 2005. **102**(32): p. 11480-5.
137. Shi, Y., et al., *Glia maturation factor gamma (GMFG): a cytokine-responsive protein during hematopoietic lineage development and its functional genomics analysis.* Genomics Proteomics Bioinformatics, 2006. **4**(3): p. 145-55.
138. Pina, C., et al., *MLLT3 regulates early human erythroid and megakaryocytic cell fate.* Cell Stem Cell, 2008. **2**(3): p. 264-73.
139. Cao, W., et al., *CXXC finger protein 1 is critical for T-cell intrathymic development through regulating H3K4 trimethylation.* Nat Commun, 2016. **7**: p. 11687.
140. Nemeth, M.J., et al., *Hmgb3 deficiency deregulates proliferation and differentiation of common lymphoid and myeloid progenitors.* Blood, 2005. **105**(2): p. 627-34.
141. Gosling, K.M., et al., *Defective T-cell function leading to reduced antibody production in a kleisin-beta mutant mouse.* Immunology, 2008. **125**(2): p. 208-17.
142. Nasta, F., et al., *Increased Foxp3+ regulatory T cells in poly(ADP-Ribose) polymerase-1 deficiency.* J Immunol, 2010. **184**(7): p. 3470-7.
143. Kishi, Y., et al., *Protein C receptor (PROCR) is a negative regulator of Th17 pathogenicity.* J Exp Med, 2016. **213**(11): p. 2489-2501.
144. Woolf, E., et al., *Runx3 and Runx1 are required for CD8 T cell development during thymopoiesis.* Proc Natl Acad Sci U S A, 2003. **100**(13): p. 7731-6.
145. Wheat, J.C., et al., *The corepressor Tle4 is a novel regulator of murine hematopoiesis and bone development.* PLoS One, 2014. **9**(8): p. e105557.
146. Chaudhuri, L., et al., *Uncoupling protein 2 regulates metabolic reprogramming and fate of antigen-stimulated CD8+ T cells.* Cancer Immunol Immunother, 2016. **65**(7): p. 869-74.
147. Barshack, I., et al., *The loss of calretinin expression indicates aganglionosis in Hirschsprung's disease.* J Clin Pathol, 2004. **57**(7): p. 712-6.
148. Roney, K.E., et al., *Plexin-B2 negatively regulates macrophage motility, Rac, and Cdc42 activation.* PLoS One, 2011. **6**(9): p. e24795.
149. Alves, M.M., et al., *Mutations in SCG10 are not involved in Hirschsprung disease.* PLoS One, 2010. **5**(12): p. e15144.
150. Kimmins, S., et al., *Differential functions of the Aurora-B and Aurora-C kinases in mammalian spermatogenesis.* Mol Endocrinol, 2007. **21**(3): p. 726-39.
151. Meehan, T., et al., *Developmental regulation of the bcl-2 family during spermatogenesis: insights into the sterility of bcl-w-/- male mice.* Cell Death Differ, 2001. **8**(3): p. 225-33.
152. Kaushal, N. and M.P. Bansal, *Inhibition of CDC2/Cyclin B1 in response to selenium-induced oxidative stress during spermatogenesis: potential role of Cdc25c and p21.* Mol Cell Biochem, 2007. **298**(1-2): p. 139-50.
153. Nah, W.H., et al., *Claudin-11 expression increased in spermatogenic defect in human testes.* Fertil Steril, 2011. **95**(1): p. 385-8.
154. Siep, M., et al., *Basic helix-loop-helix transcription factor Tcfl5 interacts with the Calmegin gene promoter in mouse spermatogenesis.* Nucleic Acids Res, 2004. **32**(21): p. 6425-36.
155. Liu, Y.L., et al., *OSR1 and SPAK cooperatively modulate Sertoli cell support of mouse spermatogenesis.* Sci Rep, 2016. **6**: p. 37205.

156. Zhao, S., et al., *PDZ-binding kinase participates in spermatogenesis.* Int J Biochem Cell Biol, 2001. **33**(6): p. 631-6.
157. Burk, R.F. and K.E. Hill, *Selenoprotein P-expression, functions, and roles in mammals.* Biochim Biophys Acta, 2009. **1790**(11): p. 1441-7.
158. Tanwar, P.S., et al., *Altered LKB1/AMPK/TSC1/TSC2/mTOR signaling causes disruption of Sertoli cell polarity and spermatogenesis.* Hum Mol Genet, 2012. **21**(20): p. 4394-405.
159. Wang, Y.L., et al., *Overexpression of ubiquitin carboxyl-terminal hydrolase L1 arrests spermatogenesis in transgenic mice.* Mol Reprod Dev, 2006. **73**(1): p. 40-9.
160. Troy, T.C. and K. Turksen, *The targeted overexpression of a Claudin mutant in the epidermis of transgenic mice elicits striking epidermal and hair follicle abnormalities.* Mol Biotechnol, 2007. **36**(2): p. 166-74.
161. Shimomura, Y., et al., *Characterization of human keratin-associated protein 1 family members.* J Investig Dermatol Symp Proc, 2003. **8**(1): p. 96-9.
162. Lien, W.H., et al., *In vivo transcriptional governance of hair follicle stem cells by canonical Wnt regulators.* Nat Cell Biol, 2014. **16**(2): p. 179-90.

Supplementary Figure S6. Example images for quantification used in Figure 2B, C.
Figure shows CHH case 2 cells (top) and Control 2 cells (bottom), fixed at the indicated time after an EdU pulse. Images show DAPI staining (left), EdU staining (middle), and merge (right).

Case 2

DAPI EdU Merge

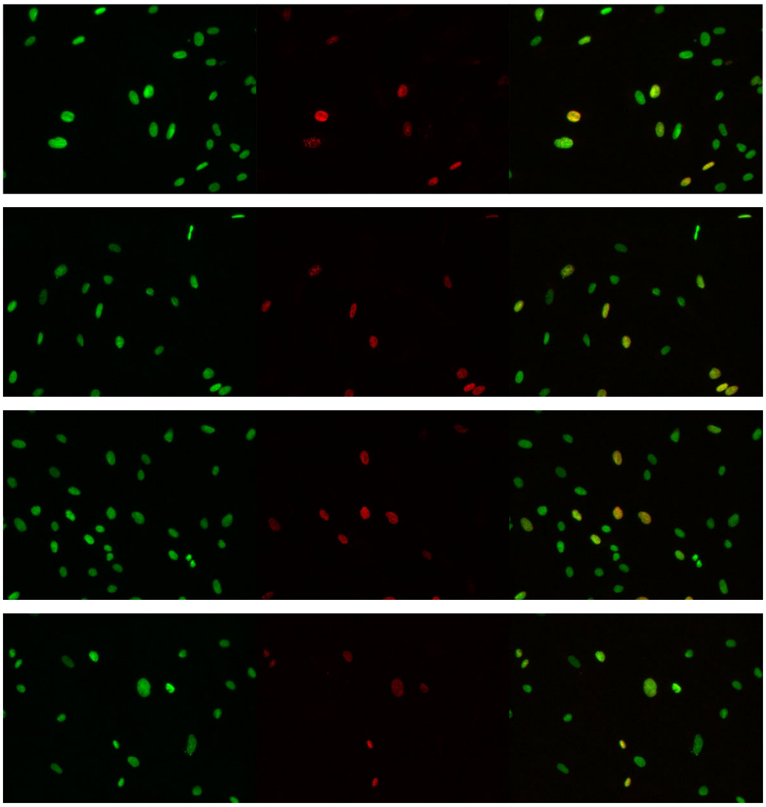

0h

5h

7.5h

10h

Control 2

DAPI EdU Merge

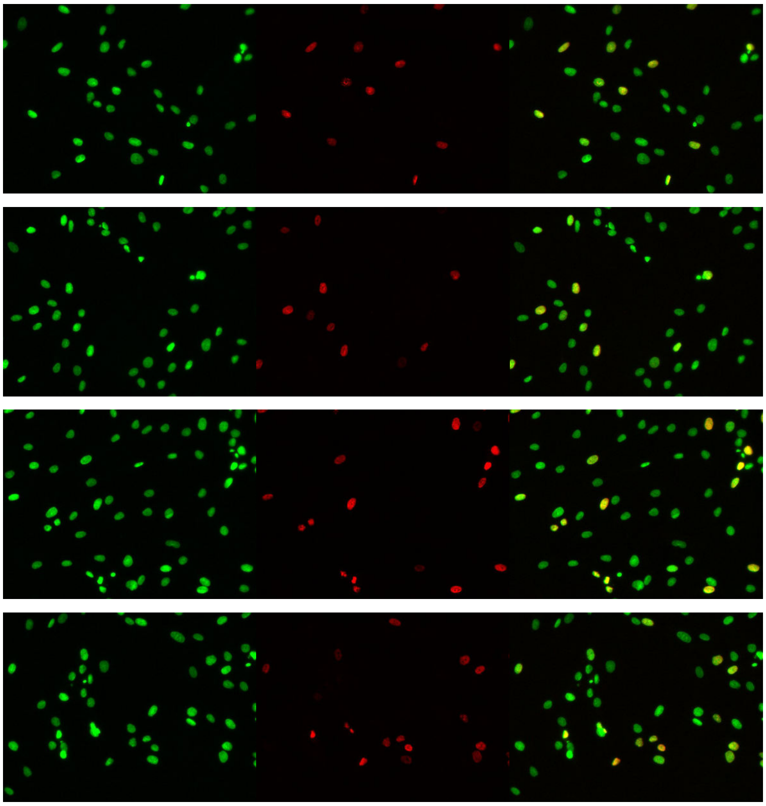

0h

5h

7.5h

10h
